# Uncovering the role of FOXA2 in the Development of Human Serotonin Neurons

**DOI:** 10.1002/advs.202303884

**Published:** 2023-09-07

**Authors:** Ting Xu, Lining Cao, Jinjin Duan, Yingqi Li, You Li, Zhangsen Hu, Shuanqing Li, Meihui Zhang, Guanhao Wang, Fei Guo, Jianfeng Lu

**Affiliations:** ^1^ Shanghai YangZhi Rehabilitation Hospital (Shanghai Sunshine Rehabilitation Center) Frontier Science Center for Stem Cell Research School of Life Sciences and Technology Tongji University Shanghai 200092 China; ^2^ Key Laboratory of Receptor Research Shanghai Institute of Materia Medica Chinese Academy of Sciences Shanghai 201203 China; ^3^ Suzhou Institute of Tongji University Suzhou 215101 China

**Keywords:** FOXA2, human pluripotent stem cells, RA signaling, serotonin neurons, SHH signaling

## Abstract

Directed differentiation of serotonin neurons (SNs) from human pluripotent stem cells (hPSCs) provides a valuable tool for uncovering the mechanism of human SN development and the associated neuropsychiatric disorders. Previous studies report that FOXA2 is expressed by serotonergic progenitors (SNPs) and functioned as a serotonergic fate determinant in mouse. However, in the routine differentiation experiments, it is accidentally found that less SNs and more non‐neuronal cells are obtained from SNP stage with higher percentage of FOXA2‐positive cells. This phenomenon prompted them to question the role of FOXA2 as an intrinsic fate determinant for human SN differentiation. Herein, by direct differentiation of engineered hPSCs into SNs, it is found that the SNs are not derived from FOXA2‐lineage cells; FOXA2‐knockout hPSCs can still differentiate into mature and functional SNs with typical serotonergic identity; FOXA2 overexpression suppresses the SN differentiation, indicating that FOXA2 is not intrinsically required for human SN differentiation. Furthermore, repressing FOXA2 expression by retinoic acid (RA) and dynamically modulating Sonic Hedgehog (SHH) signaling pathway promotes human SN differentiation. This study uncovers the role of FOXA2 in human SN development and improves the differentiation efficiency of hPSCs into SNs by repressing FOXA2 expression.

## Graphical abstract

By direct differentiation of engineered human pluripotent stem cells (hPSCs) into serotonin neurons (SNs), this study reveals that human SNs are not derived from FOXA2‐lineage cells; FOXA2‐knockout hPSCs can still differentiate into mature and functional SNs; FOXA2‐overexpression suppresses SN differentiation. This study uncovers the role of FOXA2 in human SN development and improves the differentiation efficiency toward SNs by repressing FOXA2 expression.

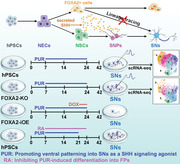

## 1 Introduction

Dysfunction of the serotonergic system have been associated with various of neuropsychiatric disorders, including depression, schizophrenia, and chronic neuropathic pain.^[^ ^1^, ^2^ ^]^ However, the fundamental mechanisms for these serotonin‐associated disorders are unclear. Human pluripotent stem cells (hPSCs)‐derived serotonin neurons (SNs) can be used for disease modeling to elucidate the pathogenesis of the diseases linked with serotonergic dysregulation and to develop novel therapeutic strategies.^[^ ^3^, ^4^ ^]^ Based on the insight into the gene‐regulation networks, we and others have successfully directed hPSCs to differentiate into SNs, which greatly facilitates the investigation of SNs on the human genetic background.^[^ ^4^, ^5^, ^6^, ^7^ ^]^ However, the contamination by undefined non‐neuronal cells in the SN differentiation system would affect the yields of SNs and make undesirable noise for data collection and analysis.

In mice, FOXA2 is believed as a key transcription factor during SN development: FOXA2‐null mice could not survive before the arise of SNs at E11.5;^[^ ^8^ ^]^ when FOXA2 was deleted in the p3 domain of r1 hindbrain region by E9.5 in the FOXA2‐Wnt1 conditional knockout (CKO) mice (Wnt1‐Cre/+; FOXA2^flox/flox^), SNs in r1 were found to be lost at E11.5.^[^ ^9^ ^]^ According to the study from Jacob et al, FOXA2 was considered to be expressed by serotonergic progenitors (SNPs) and functioned as a cell‐intrinsic factor required for SNPs to specify SN fate in mice.^[^ ^9^ ^]^ Therefore, most of the researchers including our group previously considered FOXA2 as a positive signal for the fate determination of human SNs: 1) In 2016, Xu et al. reported that human fibroblasts were transdifferentiated into SNs by lentivirus‐mediated expression of Ascl1, FOXA2, Lmx1b, and FEV, and no SNs were generated after FOXA2 was removed;^[^ ^10^ ^]^ 2) In the same year, Lu et al. suggested that increasing the proportion of FOXA2‐positive cells at SNP stage might help to obtain SNs;^[^ ^5^ ^]^ 3) The recent SN differentiation protocol from Valiulahi et al. showed that the percentage of FOXA2‐positive cells reached to 80–90% at neural progenitor stage.^[^ ^11^ ^]^ However, in our recent routine differentiation experiments for SNs, we accidentally found that less SNs and more non‐neuronal cells were obtained from SNP stage with higher percentage of FOXA2‐positive cells. Obviously, the role of FOXA2 as an intrinsic fate determinant for human SN differentiation is questionable. Rowing harder doesn't help if the boat is headed in the wrong direction. So, it is worth to revisit and uncover the role of FOXA2 in the development of human SNs, which would facilitate the development of a more efficient approach to obtain human SNs from hPSCs.

Herein, we explored the role of FOXA2 in human SN development through direct differentiation of FOXA2‐lineage‐tracing, FOXA2 knockout (FKO) or inducible FOXA2‐overexpression (FOXA2‐iOE) hPSCs into SNs. We found that the SNs were not derived from FOXA2‐lineage cells; FKO‐hPSCs could still differentiate into mature and functional SNs with typical serotonergic identity; FOXA2 overexpression suppressed the SN differentiation, indicating that FOXA2 is not intrinsically required for human SN differentiation. Furthermore, repressing FOXA2 expression by retinoic acid (RA) and dynamic activation of Sonic Hedgehog (SHH) signaling pathway promoted human SN differentiation. This study uncovers the role of FOXA2 in human SNs development and improves the differentiation efficiency of hPSCs into SNs by repressing FOXA2 expression.

## 2 Results and Discussion

### 2.1 Human SNs are not Derived from FOXA2‐Positive Cells

**Figure 1:**
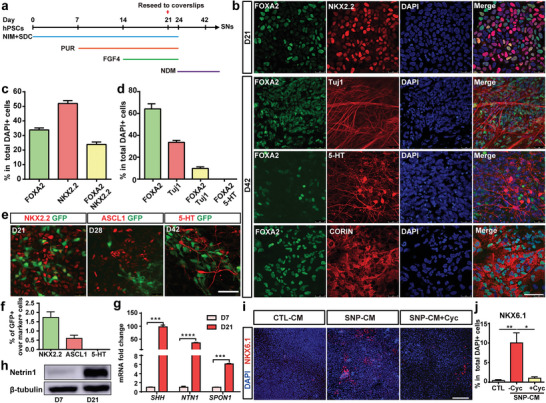
Identification of FOXA2‐positive cells during human SN differentiation. a) Schematic of SN differentiation protocol. b) Immunofluorescence staining for cells at day 21 (SNP stage) and day 42 (SN stage). c, d) Quantification for b. e) Immunofluorescence staining for cells at 3 key stages of differentiation (day 21, 28, and 42) of FOXA2‐lineage‐tracing hPSCs toward SNs. f) Quantification for e. g) mRNA expression levels for SHH, Netrin1 (*NTN1*), and F‐Spondin (*SPON1*). h) Western blotting of Netrin1. i) Immunofluorescence staining of NKX6.1 after 7 days in CM cultures. j) Quantification for i. Data are represented as mean ± SEM from three independent experiments. *p < 0.05; **p < 0.01; ***p < 0.001; ****p < 0.0001. hPSCs: human pluripotent stem cells; NIM: neural induction medium; SDC: SB431542, DMH1, CHIR99021; PUR: purmorphamine; NDM: neural differentiation medium; CTL: control; SNP: serotonergic progenitor; CM: conditioned medium (supernatant); Cyc: cyclopamine. Scale bar: (b, e) 50 µm; (i) 100 µm.

SN differentiation was carried out as we previously described with some modifications.^[^ ^5^ ^]^ Purmorphamine (PUR, a SHH signaling agonist) was used to activate SHH signaling pathway (**Figure** **1**a). At day 21 (SNP stage), ≈30% of the differentiated cells expressed FOXA2; ≈60% of the cells expressed NKX2.2 (a marker for SNP); but only ≈24% of the cells co‐expressed FOXA2 and NKX2.2 (Figure 1b,c). At day 42 (SN stage), the FOXA2‐positive cells expanded to >60%, most of which showed non‐neuronal morphology with only 10% expressed the neuronal marker Tuj1. Moreover, the 5‐HT‐positive SNs were negative for FOXA2 (Figure 1b,d). This prompted us to question whether SNs are derived from FOXA2‐positive cells and the role of FOXA2 in specifying serotonergic fate.

To determine whether human SNs are derived from FOXA2‐positive cells, a FOXA2 lineage‐tracing hPSC line was differentiated into SNs. This FOXA2 lineage‐tracing system enables us to mark cells who ever express FOXA2 with GFP, which could be retained permanently and passed on to all progeny of the founder cell.^[^ ^12^ ^]^ At day 21, 28, and 42 of differentiation toward SNs, the non‐specific serotonergic markers (NKX2.2 and SOX1 for SNPs; ASCL1 a proneural gene for postmitotic serotonergic precursors;^[^ ^13^ ^]^ GATA3 for mature SN) and the specific serotonergic markers (5‐HT and TPH2) were investigated, respectively. More than 98% of cells expressing NKX2.2, ASCL1, SOX1, or GATA3 did not exhibit co‐staining with GFP, while only 0.5%−2% of them displayed co‐staining with GFP (Figure 1e,f; Figure S1a,b, Supporting Information). It is reasonable to speculate that the co‐labeling of GFP with a non‐specific serotonergic marker may indicate other cell types instead of SNs, since these markers are not specific for serotonergic cells. Actually, NKX2.2 is not only expressed in SNPs but also in the early‐stage floor plate (FP) cells;^[^ ^14^ ^]^ progenitors expressing ASCL1 or SOX1 have the potential to differentiate into multiple neuronal types within the brain;^[^ ^15^, ^16^ ^]^ GATA3 is also expressed in auditory neurons.^[^ ^17^ ^]^ Consistent with our speculation, no GFP‐positive cell was co‐stained with 5‐HT or TPH2 (the specific markers for mature SNs) (Figure 1e,f; Figure S1a,b, Supporting Information), indicating that SNs were not derived from FOXA2‐positive cells.

Then we investigated the identity of FOXA2‐positive cells. Quantitative PCR (qPCR) data showed that the differentiated cells (at day 21) expressed FP cell specific markers SHH, Netrin1 (NTN1) and F‐Spondin (SPON1) (Figure 1g). Western blotting data confirmed the expression of Netrin1 (Figure 1h). At day 42, the majority (>90%) of FOXA2‐positive cells were co‐stained with CORIN, a FP cell marker (Figure 1b). Functional assays were conducted to verify the identity of FOXA2‐positive cells as FP cells. One of the functional features for FP cells is their ability to secrete SHH, a potent ventralization factor.^[^ ^18^ ^]^ At day 21, PUR and FGF4 were withdrawn from the medium and the supernatant of differentiated cells was collected at day 24 (the supernatant of the FOXA2‐negative cells was collected at day 24 as a negative control for SHH secretion, Figure S1c, Supporting Information). The supernatant of FOXA2‐positive cells could ventralize the neuroepithelium cells to express the ventral marker NKX6.1, while cyclopamine (Cyc, a SHH signaling pathway antagonist) inhibited the ventralization (Figure 1i,j). These data suggested that FOXA2‐positive cells might be non‐neuronal FP cells instead of SNPs or SNs.

### 2.2 FKO hPSCs are Able to Differentiate into Functional SNs

**Figure 2:**
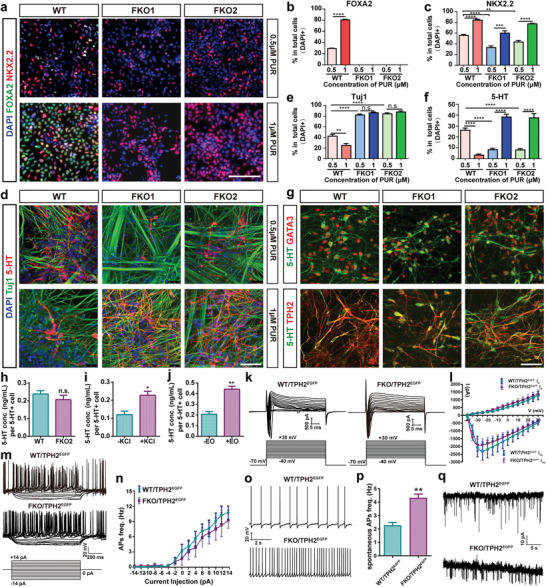
FKO hPSCs could differentiate into SNs. a) Immunofluorescence staining for cells treated with lPUR (0.5 µM) and hPUR (1 µM) at day 21 of differentiation. b, c) Quantification for a. d) Immunofluorescence staining for cells treated with lPUR and hPUR at day 42 of differentiation. e, f) Quantification for d. g) Immunofluorescence staining for cells at day 42 of differentiation (treatment with lPUR for WT cells and hPUR for FKO cells). h) Extracellular 5‐HT released by WT‐ and FKO‐SNs after 1‐h incubation in fresh NDM. i, j) Extracellular 5‐HT released by FKO‐SNs before and after stimulation by KCl (i) or EO (j). k) Representative current traces evoked by 40‐ms depolarizing voltages stepped from −40 to +30 mV in 5 mV increments (upper) and input voltage program (lower) for WT‐TPH2^EGFP^‐ or FKO2‐TPH2^EGFP^‐SNs. l) Current‐voltage curves for voltage‐gated Na+ and K+ currents of WT‐TPH2^EGFP^‐SNs (n = 6) and FKO2‐TPH2^EGFP^‐SNs (n = 5). m) Schematic of current stimulation‐induced APs for WT‐TPH2^EGFP^‐SNs (n = 6) or FKO‐TPH2^EGFP^‐SNs (n = 5) and input current program. n) Quantification of the AP frequency triggered by input current from −14 pA to +14 pA (n = 5 in FKO group; n = 6 in WT group). o) Representative traces of spontaneous APs generated by WT‐TPH2^EGFP^‐SNs (n = 3) or FKO‐TPH2^EGFP^‐SNs (n = 3). p) Quantification for the frequency of spontaneous APs generated by the 2 groups of cells (n = 3). q) Representative traces of sEPSCs generated by WT‐TPH2^EGFP^‐SNs (n = 3) or FKO‐TPH2^EGFP^‐SNs (n = 3). Data are represented as mean ± SEM from three independent experiments. **p < 0.01; ***p < 0.001; ****p < 0.0001; n.s.: no significance. WT: wide type; conc: concentration; APs: action potentials; EO: Escitalopram Oxalate; sEPSCs: spontaneous excitatory postsynaptic currents. Scale bar: (a) 100 µm; (d, g) 50 µm.

To elucidate whether FOXA2 is required for serotonergic fate determination, we generated FKO hPSC lines by two strategies using CRISPR/Cas9 gene‐editing technology: 1) the stop codon sequence was inserted into the exon 2 of the FOXA2 gene, ultimately resulting in a shortened non‐functional protein (named FKO1, Figure S2a, Supporting Information); 2) an indel was introduced into the FOXA2 gene, thus leading to a frameshift mutation and a premature stop codon (named FKO2, Figure S2b, Supporting Information). FOXA2 (also known as HNF‐3β) is a direct downstream target of SHH signaling pathway,^[^ ^19^ ^]^ thus we activated SHH signaling pathway by PUR to assess whether FOXA2 was knocked out in FKO cells. Wild‐type (WT) cells and the two FKO hPSC lines were differentiated into neural stem cells (NSCs), then PUR was added to induce FOXA2 expression. FOXA2 was expressed in WT cells but not the FKO cells (**Figure** **2**a,b; Figure S2c, Supporting Information), indicating that FOXA2 was successfully knocked out in the edited cell lines.

WT and FKO human embryonic stem cells (hESCs, H9) were then differentiated into SNs according to the protocol (Figure 1a). The cells were treated with low concentration of PUR (0.5 µM, lPUR), at day 21 of differentiation (SNP stage), the percentage of NKX2.2‐positive cells in FKO groups was lower than that in WT group (Figure 2a,c). This might be caused by the lack of FOXA2‐positive FP cells in FKO groups to secrete SHH, which is well‐known to promote NKX2.2 expression.^[^ ^20^ ^]^ We thus increased the concentration of PUR to 1 µM (high concentration of PUR, hPUR) and identified that hPUR‐treatment increased the percentage of NKX2.2‐positive cells in both WT and FKO groups, while hPUR‐treatment increased the percentage of FOXA2‐positive cells only in WT group (Figure 2a–c). At day 42 (SN stage), lPUR‐treatment significantly increased the percentage of Tuj1‐positive neurons in the FKO groups when compared to WT group, while hPUR‐treatment decreased the percentage of Tuj1‐positive neurons only in WT group but not in FKO groups (Figure 2d,e). When treated with lPUR, the percentage of 5‐HT‐positive SNs in the FKO group was significantly lower than that in WT group (Figure 2d,f). Conversely, when treated with hPUR, the proportion of 5‐HT‐positive SNs exhibited a significant increase in the FKO groups compared to WT group, indicating that increasing the concentration of PUR could rescue the lower percentage of SNs derived from FKO cells. It is notable that increasing the concentration of PUR led to a significant decrease in the proportion of 5‐HT‐positive SNs in WT group (Figure 2d,f). These data supported our speculation that the increase of FOXA2‐positive cells negatively affected the differentiation toward SNs. In addition, FKO hPSCs‐derived SNs (FKO‐SNs) also expressed other markers for mature SNs: GATA3 and TPH2 (Figure 2g). FKO cells derived from two other hPSC lines (a hESC line‐H1 and a human induced pluripotent stem cell [iPSC] line‐ZSSY001) also could be differentiated into SNs (Figure S2d,e, Supporting Information). To evaluate the influence of hPUR (1 µM) treatment on the ratios of other neurons in the differentiation culture, qPCR and immunofluorescence staining were performed to detect the markers for glutamatergic, GABAergic, cholinergic, noradrenergic or dopaminergic neurons. Compared to lPUR‐treatment, hPUR induced a decrease in the expression of cholinergic markers in FKO‐group but inconsistent influence on the expression of markers for glutamatergic and GABAergic neurons in WT‐ and FKO‐groups (Figure S3a–f, Supporting Information). Obviously, however, hPUR treatment significantly increased the proportion of TH‐positive noradrenergic or dopaminergic neurons in both WT and FKO cells when compared to lPUR‐treated cells (Figure S3g–j, Supporting Information). Indeed, SHH signaling is crucial for the development of noradrenergic or dopaminergic neurons.^[^ ^21^, ^22^ ^]^ These data suggested that FKO hPSCs could differentiate into mature SNs and the limited differentiation potential of FKO cells toward SNs might be due to the lack of FP cells to secrete SHH. Therefore, instead of obtaining high percentage of FOXA2 cells, sufficient activation of SHH signaling without increasing FOXA2‐positive cells is crucial for the efficient SNs differentiation. FOXA2 is not intrinsically required for human SN differentiation.

5‐HT secretion capacity of FKO‐SNs was investigated by examining the incubation medium with enzyme linked immunosorbent assay (ELISA). Compared to WT‐SNs, FKO‐SNs showed similar ability to secret 5‐HT (Figure 2h), which could be stimulated by high concentration of potassium ion and Escitalopram Oxalate (EO, a selective serotonin reuptake inhibitor, SSRI) (Figure 2i,j). Therefore, the FKO‐SNs showed the normal capability to synthesize and release 5‐HT.

To facilitate the examination of the electrophysiological properties of SNs, a TPH2^EGFP^ reporter system was constructed into the TPH2 locus to indicate TPH2‐positive SNs with EGFP as we described previously.^[^ ^23^ ^]^ The whole‐cell patch‐clamp recording was applied. The voltage clamp was used to generate stepped membrane voltages, and the current flowing through the membrane was measured. The patched FKO‐SNs showed typical traces of currents similar to WT‐SNs (Figure 2k). The inward sodium current (I_Na_) or the outward potassium current (I_K_) evoked by a fixed voltage was comparable between WT‐ and FKO‐SNs (Figure 2l). The current clamp was used to elicit action potential (APs) by injecting current into SNs. A WT‐SN or an FKO‐SN fired trains of APs under the current clamp (Figure 2m). The frequency of the evoked APs of WT‐ and FKO‐SNs was comparable (Figure 2n). Compared to spontaneous APs generated by WT‐SNs (2.28 ± 0.36 Hz, n = 3), the FKO‐SNs showed a significantly higher frequency of spontaneous APs (4.325 ± 0.2758 Hz, n = 3) (Figure 2o,p), which may be caused by the different cell composition in the culture of WT‐SNs and FKO‐SNs (more Tuj1‐positive neurons but no FOXA2‐positive non‐neuronal cells). Spontaneous excitatory postsynaptic currents (sEPSCs) were detected to evaluate synaptic transmission. Similar with WT‐SNs, FKO‐SNs also exhibited sEPSCs (Figure 2q), indicating the formation of a functional synaptic network between the FKO‐SNs and surrounding cells. Above all, FKO‐SNs exhibited functional 5‐HT secretion capacity and electrophysiological properties, suggesting that FOXA2 is not intrinsically required for the functions of human SNs.

### 2.3 Subpopulation Classification and Transcriptome Profiles of FKO‐SNs

To fully understand the influence of FOXA2‐knockout on SN differentiation, single‐cell RNA‐sequencing (scRNA‐seq) was performed to compare the subpopulation classification and transcriptomic profiles of WT‐ and FKO‐SNs. TPH2^EGFP^ reporter system was applied to purify SNs for scRNA‐seq with desired sequencing depth.

**Figure 3:**
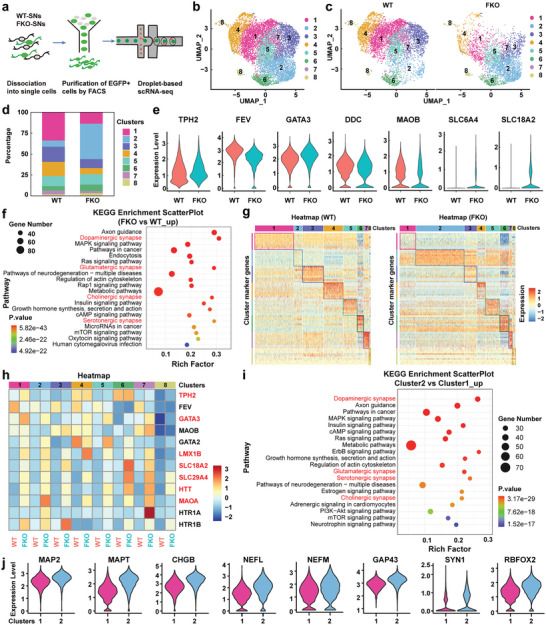
Comparative analysis of subpopulation composition and transcriptomic profiles of WT‐ and FKO‐SNs. a) Schematic of scRNA‐seq based on FACS‐purified TPH2^EGFP^‐SNs. b) Integration of the scRNA‐seq datasets of WT‐ and FKO‐SNs. c) Split UMAP plots for WT‐ and FKO‐SNs. d) The proportion of each cluster in WT‐ and FKO‐SNs. e) Violin plots showing the expression levels of serotonergic marker genes in WT‐ and FKO‐SNs after integration. f) KEGG enrichment analysis of the upregulated DEGs in FKO‐SNs. g) Heatmap of top 10 marker genes within each cluster using scRNA‐seq. The top marker genes were selected using the bimod test and ranked based on their Log2FC value within each identified cluster. In both panels, rows correspond to genes and columns to clusters (subpopulations). h) Heatmap of the serotonergic‐related genes. i) KEGG enrichment analysis of the upregulated DEGs in cluster 2. j) Violin plots showing the expression level of mature neuronal markers. MAP2: microtubule‐associated protein 2; MAPT: MAP‐tau; CHGB: chromogranin B, NEFL, NEFM: neurofilament light/medium chain; GAP43: growth associated protein 43, SYN1: synapsin 1; RBFOX2: RNA Binding Fox‐1 Homolog 2.

At day 42 of differentiation, fluorescence‐activated cell sorting (FACS) was performed to purify SNs (**Figure** **3**a). After a stringent filtering‐out step, 3891 and 6000 high‐quality single cells from FKO and WT groups respectively, were obtained and used for further analysis. To compare the WT‐ and FKO‐SNs, we combined the scRNA‐seq datasets of the two samples (Figure 3b; Figure S4a,b, Supporting Information). We found that the UMAP clustering on FKO‐SNs gave clusters similar to WT‐SNs: the datasets largely overlapped and the contributions from each sample were shown in Figure 3c. Both WT‐ and FKO‐SNs can be categorized into 8 clusters, with cluster 1 being the most prominent in WT‐SNs and cluster 2 being the most prevalent in FKO‐SNs (Figure 3d). The expression levels of the key serotonergic markers were similar in both groups (Figure 3e; Figure S4c, Supporting Information). All of the WT‐ and FKO‐SNs expressed TPH2, and the majority of cells express FEV, GATA3, DDC, and MAOB (Figure S4c, Table S1, Supporting Information), indicating their identity as mature SNs. Furthermore, some WT‐ and FKO‐SNs even expressed the two typical mature SN markers SLC6A4 (SERT) and SLC18A2 (VMAT2) (Figure 3e, Figure S4c, Table S1, Supporting Information), which were also reported in the scRNA‐seq data of in vivo mouse SNs.^[^ ^24^ ^]^ This data validated the serotonergic identity of FKO‐SNs.

A comprehensive comparative analysis of the transcriptomic profiles between WT and FKO groups were also conducted. First, differentially expressed genes (DEGs) were analyzed. FKO‐SNs exhibited 656 downregulated DEGs and 1710 upregulated DEGs (Figure S4d, Supporting Information), which were highly enriched in the synapse‐associated KEGG pathways (Figure 3f), indicating an improved synaptic function and enhanced neuronal maturity of FKO‐SNs. The top 5 upregulated‐ and downregulated‐ genes in each cluster of FKO‐SNs were listed in the volcano plots (Figure S4e, Supporting Information). Then the combined datasets were split to assess the expression levels of the top 10 marker genes within each cluster of the two groups. Although WT‐ and FKO‐SNs showed similar expression levels of marker genes in cluster 3–8, the FKO‐SNs exhibited higher expression levels of cluster 2 marker genes and lower expression levels of cluster 1 marker genes when compared to those of WT‐SNs (Figure 3g). Subsequently, KEGG enrichment analysis was performed to identify the functional annotation of the marker genes of cluster 1 and 2 (Figure S4f,g, Supporting Information). The marker genes in cluster 2 were identified to be enriched in synaptic‐related pathways (Figure S4g, Supporting Information). Although there were differences in the expression levels of marker genes within cluster 1 and cluster 2 between the two groups, each cluster of FKO‐SNs exhibited higher expression levels of the 7 serotonergic‐related genes (marked in red) (Figure 3h, Table S2, Supporting Information). Because cluster 1 and cluster 2 respectively represented the prominent subpopulations of WT‐ and FKO‐SNs, the DEGs between the 2 clusters were investigated. Compared to cluster 1, cluster 2 showed 1152 upregulated DEGs (Figure S4h, Supporting Information), which were highly enriched in synapse‐related KEGG pathways (Figure 3i). Moreover, the cluster 2 showed higher expression levels of the genes commonly used to indicate mature neurons (Figure 3j).^[^ ^25^, ^26^, ^27^, ^28^, ^29^, ^30^ ^]^ These data indicated that FKO‐SNs not only showed typical serotonergic identity and similar subpopulation classification as WT‐SNs, but also a more advanced degree of maturation compared to WT‐SNs.

During neuronal development, neurons undergo a sequence of molecular and structural alterations that culminate in the formation of functional synapses at the mature stage. Therefore, the presence of the synaptic‐associated DEGs indicates more efficient synaptic transmission, neuronal communication and overall synaptic function. The upregulation of the synaptic‐associated DEGs in FKO‐SNs and the cluster 2 (the predominant subpopulation of FKO‐SNs) (Figure 3f,i) suggested that the FKO‐SNs reached a more advanced maturation stage where they possess a more robust synaptic function. This might be explained by the increased proportion of 5‐HT‐positive SNs in the medium of FKO groups (Figure 2d,f), which might subsequently contribute to the production of higher level of 5‐HT in the medium. Although 5‐HT is well‐known as a crucial developmental signal which influences neuronal developmental process from neurogenesis, axon guidance, dendritic growth to synapse formation,^[^ ^31^ ^]^ there is limited direct evidence regarding the effects of 5‐HT on the development of SNs themselves. The specific influence of 5‐HT on SNs maturation requires further investigation.

To further validate the identity of the human FKO‐SNs in vitro, our data were compared with a 10X single‐cell transcriptomics dataset of in vivo mouse SNs.^[^ ^32^ ^]^ The mouse‐ and human‐SNs showed large overlap in tSNE plots (Figure S5a,b, Supporting Information), indicating the similarity of SNs at single cell level across species. The human SNs showed higher expression of the key transcription factors (FEV and GATA3) which were commonly expressed by early‐stage SNs but lower expression of 5‐HT synthesis‐ and transportation‐related genes as TPH2, DDC, SLC6A4, and SLC18A2 which were commonly expressed by SNs at the late maturation stage (Figure S5c,d, Supporting Information), indicating a lower maturation stage of in vitro human SNs when compared to in vivo mouse SNs. Subpopulation classification of SNs showed great similarity across species, the combined human and mouse datasets were classified into 23 clusters, among which 17 overlapping clusters were shared by human and mouse SNs; however, the proportion of the clusters was quite different across species (Figure S5e–g, Supporting Information). It is worth mentioning that mouse SNs had 6 unique clusters (cluster 8, 16, 17, 21, 22, and 23), with cluster 8 being the most prominent (14.27%) among the 23 clusters (Figure S5f,g, Supporting Information). We also found that cluster 8 showed high expression level of the 5 mature serotonergic markers but relatively lower expression level of the 2 early‐stage serotonergic markers (Figure S5h, Supporting Information), indicating that cluster 8 might be the subpopulation of mouse SNs at more advanced maturation stage. Based on the expression patterns of the previously reported 399 SN diversity‐associated genes (Table S3, Supporting Information) by the individual cluster,^[^ ^24^, ^32^, ^33^, ^34^, ^35^ ^]^ we then performed correlation analysis of the 23 clusters across species: the human WT‐ and FKO‐SNs showed strong correlation among clusters (Figure S5i, Supporting Information), demonstrating that FKO did not influence the serotonergic identity of the individual cluster of human SNs. It is worth mentioning that human FKO‐SNs showed higher correlation to in vivo mouse SNs when compared to human WT‐SNs (Figure S5j,k, Supporting Information), indicating an enhanced neuronal maturity of FKO‐SNs similar to in vivo mouse SNs. These data demonstrated that human and mouse SNs exhibited similarity in subpopulation classification, but the respective transcriptomic information of the shared cluster was quite different. Moreover, in vivo mouse SNs were more mature than in vitro hPSCs‐derived human SNs (Figure S5c,d, Supporting Information). This is reasonable: activation of the serotonergic gene battery only generates newborn SNs, and full maturation of SNs requires intricate developing signals,^[^ ^36^ ^]^ complicated interactions with glial cells‐ and extracellular matrix‐,^[^ ^37^, ^38^ ^]^ and integration with the complex in vivo neural circuitry.^[^ ^39^ ^]^ It is challenging to mimic the in vivo environment precisely in an in vitro setting.

### 2.4 FOXA2 Overexpression Suppresses SN Differentiation

**Figure 4:**
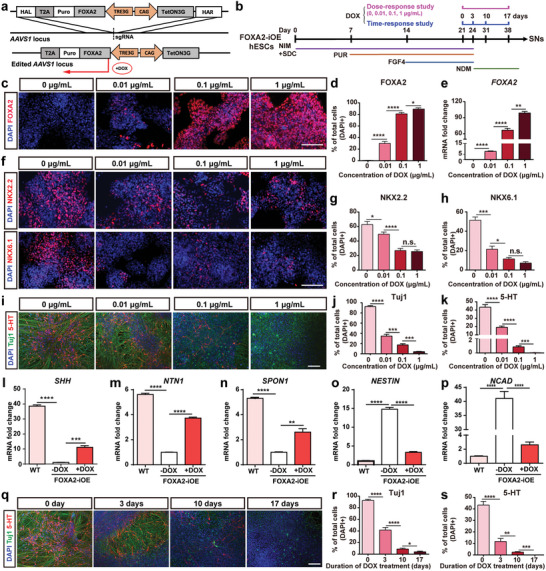
FOXA2 overexpression promotes FP cell differentiation but represses SN differentiation. a) Gene‐editing strategy for the construction of FOXA2‐iOE hPSC line. b) Schematic of SNs differentiation protocol and DOX‐treatment strategy. c–h) Immunofluorescence staining and quantification of FOXA2+ cells (c, d), NKX2.2+ cells (f, g) and NKX6.1+ cells (f, h), and FOXA2 mRNA expression level (e) of FOXA2‐iOE cells in response to increasing concentrations of DOX for 3 days. i–k) Immunofluorescence staining and quantification of Tuj1+ cells (i, j) and 5‐HT+ cells (i, k) of FOXA2‐iOE cells in response to increasing concentrations of DOX. for 17 days. l–p) mRNA expression levels of SHH l), NTN1 (m), SPON1 (n), NESTIN (o) and NCAD (p) at day 24 of differentiation with or without DOX‐treatment for three days. q–s) Immunofluorescence staining and quantification of Tuj1+ cells (q, r) and 5‐HT+ cells (q, s) at day 38 of differentiation after DOX‐treatment for different duration. Data are represented as mean ± SEM from three independent experiments. *p < 0.05; **p < 0.01; ***p < 0.001; ****p < 0.0001; n.s.: no significance. DOX: doxycycline; WT: wide type. Scale bar: (c, f, i, q) 100 µm.

To elucidate whether FOXA2 overexpression gives rise to the large portion of non‐neuronal cells during SN differentiation, FOXA2‐iOE hPSC line was generated on the genetic background of FKO2 (**Figure** **4**a). FOXA2 overexpression was driven by doxycycline (DOX) (Figure S6, Supporting Information). The dose‐response and time‐response studies of DOX on FOXA2 overexpression and SN differentiation were designed and conducted (Figure 4b). For the dose‐response study: at day 21 of differentiation, the cells were treated with increasing concentrations of DOX (0, 0.01, 0.1, 1 µg mL^−1^) (Figure 4b). A positive correlation between the concentrations of DOX and the expression of FOXA2 was observed after 3 days DOX treatment (Figure 4c–e). More importantly, with the increase of DOX concentration, there was a corresponding decrease in the expression of SNP markers (NKX2.2 and NKX6.1) (Figure 4f–h), the neuronal marker (Tuj1) and serotonergic marker (5‐HT) (Figure 4i–k). These data indicated that DOX‐treatment induced FOXA2 overexpression and consequently suppressed SN differentiation in a dose‐dependent manner. For the time‐response study: at day 21 of differentiation, the FOXA2‐iOE cells were treated with DOX (1 µg mL^−1^) for 3, 10, or 17 days (Figure 4b). At day 24 of differentiation, FOXA2‐iOE cells without DOX treatment (similar to FKO cells) showed significantly decreased mRNA levels for FP cell‐specific markers (SHH, NTN1, and SPON1) and remarkably increased mRNA levels for NSC markers (NESTIN and NCAD) compared to WT cells, while FOXA2‐iOE cells treated with DOX showed increased mRNA levels of FP cell‐specific markers and decreased mRNA levels of NSC markers compared to FOXA2‐iOE cells without DOX treatment (Figure 4l–p), suggesting the involvement of FOXA2 in determining the fate toward non‐neuronal FP cells. After 2 weeks of further culture in neuron differentiation medium (NDM), DOX‐treatment effectively decreased the number of Tuj1‐positive neurons and 5‐HT‐positive SNs in a time‐dependent manner (Figure 4q–s). These data suggested that DOX‐induced FOXA2‐overexpression promoted differentiation toward non‐neuronal FP cells and suppressed differentiation into SNs.

FOXA2‐overexpression at day 21 of differentiation (SNP stage) could suppress the differentiation toward SNs. However, Xu et al. suggested that combined expression of FOXA2, Ascl1, Lmx1b, and FEV promoted the trans‐differentiation of human fibroblasts into SNs.^[^ ^10^ ^]^ This contradictory result can be explained by the different genetic methods mediated FOXA2 overexpression and the different cell types in which FOXA2 was overexpressed. In Xu's study, fibroblasts were infected by lentiviruses to induce the overexpression of FOXA2and other transcription factors, which may lead to variable lentiviral transfection efficiency. As a consequence, it is possible that the fibroblasts infected with the lentivirus carrying FOXA2 may not trans‐differentiate into SNs but help the neighbor FOXA2‐negative cells convert into SNs by FOXA2‐mediated SHH regulation.^[^ ^40^ ^]^ Whereas the homogeneous FOXA2‐iOE line with FKO genetic background was used in our study and FOXA2‐overexpression was directly induced in the cells with serotonergic fate. Thus, FOXA2‐overexpression converted the serotonergic fate into FP cells (Figure 4f–p). Besides, FOXA2‐mediated serotonergic suppression effect showed obvious time‐dependent effect: the longer‐term treatment with DOX led to the lower production of Tuj1‐positive and 5‐HT‐positive SNs (Figure 4q–s). Therefore, we speculate the manner of how FOXA2 regulates SN differentiation: expression of FOXA2 in non‐serotonergic cells (including FP cells) promotes expression and release of SHH, which activates SHH signaling in serotonergic cells and facilitates the differentiation toward SNs; however, direct expression of FOXA2 in cells with serotonergic fate suppresses SN differentiation.

### 2.5 RA Represses FP Cells Differentiation and Promotes Neural Differentiation

**Figure 5:**
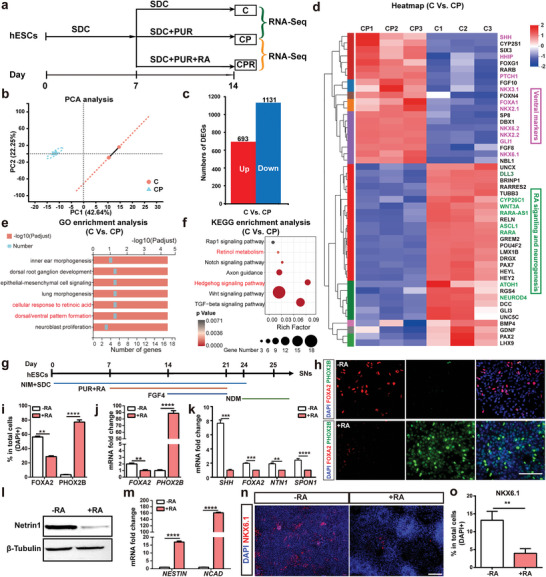
RA represses FP cell differentiation but promotes neuronal differentiation. a) Experimental design for RNA sequencing. C: treatment with SDC; CP: treatment with SDC and PUR; CPR: treatment with SDC, PUR and RA. b) PCA analysis for groups C and CP. c) Analysis of DEGs (group C vs CP). d) Heatmap of interested DEGs (group C versus CP). e, f) GO (e) and KEGG (f) enrichment analysis of the DEGs (group C versus CP). g) Schematic of the differentiation strategy with RA‐treatment. h, i) Immunofluorescence staining (h) and quantification (i) of FOXA2+ cells and PHOX2B+ cells at day 14 of differentiation. j) mRNA expression levels for FOXA2 and PHOX2B at day 14 of differentiation. k) mRNA expression levels of FP cell‐associated genes at day 21 of differentiation. l) Western blotting for Netrin1 at day 21 of differentiation. m) mRNA expression levels of NSC‐associated genes at day 21 of differentiation. n, o) Immunofluorescence staining (n) and quantification of NKX6.1+ cells (o) for NSCs after 7 days of incubation with CM. Data are represented as mean ± SEM from three independent experiments. **p < 0.01; ***p < 0.001; ****p < 0.0001. DEGs: differentially expressed genes; hESCs: human embryonic stem cells; NIM: neural induction medium; SDC: SB431542, DMH1, CHIR99021; PUR: purmorphamine; NDM: neuronal differentiation medium; CM: conditioned medium. Scale bar: (h, n) 100 µm.

SHH signaling is required to ventralize hindbrain NSCs toward serotonergic fate. However, activation of SHH signaling would also induce the development of FOXA2‐positive FP cells, which are the major unwanted cells in the SN differentiation system (Figure 1). Clarifying the molecular mechanism of SHH‐induced conversion of NSCs into the FP cells may help improve the yield of SNs. To explore SHH‐induced transcriptome changes in the hindbrain NSCs, RNA sequencing (RNA‐seq) was performed for WT hPSCs‐derived cells at day 14 of differentiation with or without PUR treatment (**Figure** **5**a). The samples were separated into two populations based on principal‐component analysis (PCA) (Figure 5b). Thousands of DEGs were identified between the two groups (Figure 5c). PUR significantly upregulated the expression of ventral markers (NKX2.1, NKX2.2, NKX6.1, and NKX6.2) and FP cell markers (SHH, HHIP, FOXA1, and GLI1), and downregulated the expression of neuron‐related genes (ASCL1, ATOH1, and NEUROD4) (Figure 5d). Then the DEGs of interest were validated using qPCR (Figure S7a,b, Supporting Information). Interestingly, the expression level of RA receptor α (RARA) was downregulated upon PUR treatment (Figure 5d). GO enrichment analysis revealed that the DEGs were significantly enriched in cellular response to RA (Figure 5e), which is consistent with the data shown in KEGG analysis (Figure 5f). These data indicated that activation of SHH signaling might affect RA signaling which is essential for neuronal differentiation. Besides, it had been reported that RA could inhibit ventralization into FOXA2‐positive FP cells without influencing NKX2.2‐positive cells in the presence of SHH.^[^ ^41^ ^]^ Therefore, we speculated that RA signaling activation might inhibit SHH‐induced FP cell differentiation and increase the proportion of SNs in the culture. Then RA was added into the neural induction medium (NIM) from day 7 to day 21 (Figure 5g). As previous studies reported the mutual repression of FOXA2 and PHOX2B (a marker of visceral motor neuron precursor, which originates from the same precursor cell population with SNs),^[^ ^9^ ^]^ we investigated their expression upon RA treatment. At day 14, the percentage of FOXA2‐positive cells was significantly reduced, while the percentage of PHOX2B‐positive cells was obviously increased in the RA‐treated group (Figure 5h,i). The expression of FOXA2 and PHOX2B was also validated by qPCR (Figure 5j). At day 21, RA treatment obviously downregulated the expression of FP cell markers (Figure 5k,l) but significantly upregulated the expression of NSC markers (Figure 5m). When incubated with the supernatant of RA‐treated cells, fewer NSCs became NKX6.1‐positive ventral neural cells (Figure 5n,o; Figure S8, Supporting Information), indicating that RA treatment decreased FOXA2‐positive cells who secret SHH. These data indicated that RA inhibited the differentiation of FOXA2‐positive FP cells and promoted neural differentiation.

### 2.6 RA Inhibits FOXA2 Expression and Promotes Caudalization Via RARα

**Figure 6:**
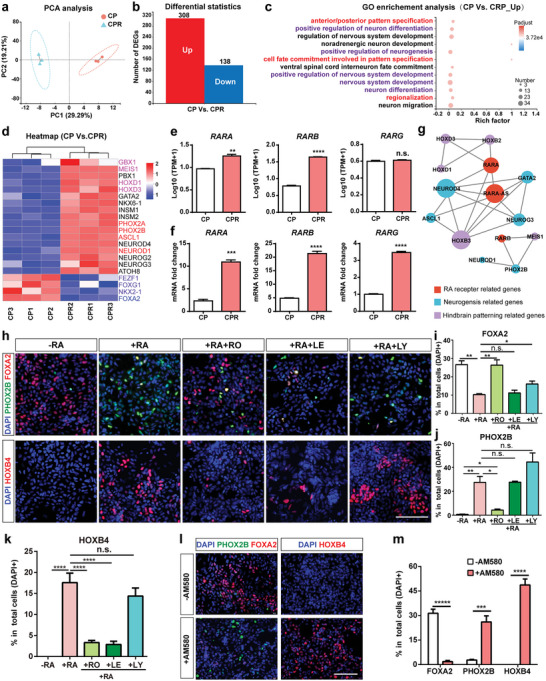
RA inhibits FOXA2 expression and promotes caudalization via RARα. a) PCA analysis for groups CP and CPR. b) Analysis of DEGs (group CP vs CPR). c) GO enrichment analysis of the DEGs (group CP versus CPR). d) Heatmap of interested DEGs (group CP vs CPR). Pink‐labeled: hindbrain‐associated genes; red‐labeled: neuron‐associated genes; purple‐labeled: forebrain‐associated genes; blue‐labeled: FP cell‐associated genes. e, f) Expression levels of RARA, RARB, and RARG derived from RNA‐seq (e) or qPCR data (f). g) Gene expression correlation of DEGs and RA receptor coding genes. h–k) Immunofluorescence staining (h) and quantification of FOXA2+ cells (i), PHOX2B+ cells (j) and HOXB4+ cells (k) at day 14 of differentiation. l, m) Immunofluorescence staining (l) and quantification (m) of FOXA2+ cells, PHOX2B+ cells and HOXB4+ cells at day 14 of differentiation with or without AM580‐treatment. Data are represented as mean ± SEM with three independent experiments. *p < 0.05; **p < 0.01; ***p < 0.001; ****p < 0.0001; n.s.: no significance. Scale bar: (h, l) 100 µm.

To elucidate the underlying mechanism by which RA inhibits FOXA2 expression, we performed RNA‐seq for hPSCs‐derived cells at day 14 of differentiation with or without RA treatment (Figure 5a). PCA separated the two groups (with RA‐treatment: group “CPR”; without RA‐treatment: group “CP”) into two distinct populations (**Figure** **6**a). RA‐treatment group showed 138 downregulated genes and 308 upregulated genes (Figure 6b), which were enriched in the categories of neuron differentiation (marked in purple) and cell fate/pattern specification (marked in red) (Figure 6c). Neurogenesis related genes (PHOX2B, PHOX2A, ASCL1, and NEUROD1) as well as hindbrain markers (GBX1, MEIS1, HOXD1, and HOXD3) were significantly upregulated in the RA‐treated cells, while FP cell‐related genes (FOXA2) and forebrain development‐related genes (FOXG1, FEZF1, and NKX2.1) were significantly downregulated (Figure 6d; Figure S7c,d, Supporting Information). In fact, apart from the newly identified inhibitory effect on FOXA2 expression, RA is a well‐documented morphogen crucial for neural caudalization.^[^ ^42^, ^43^ ^]^

Given that RA performs multiple functions by binding to distinct nuclear receptors, we aimed to identify a particular receptor which mediates RA's effect on FOXA2 repression and neural caudalization. RNA‐seq data revealed that RA‐treatment significantly increased the expression levels of RA receptors (Figure 6e), which was verified by qPCR data (Figure 6f). Gene correlation analysis indicated that the receptor RARα may play an important role in regulating RA‐mediated neurogenesis and neural caudalization (Figure 6g). Then the RA receptors inhibition/activation experiments were conducted to ascertain the role of RARα. The selective antagonists for the three RA receptors (RO 41–5253: inhibitor for RARα; LE135: inhibitor for RARβ; LY 2 955 303: inhibitor for RARγ) and RA were treated to the hPSCs‐derived NSCs at day 7 of differentiation for one week, respectively. At day 14 of differentiation, RA treatment (100 nM) significantly inhibited FOXA2 expression and upregulated PHOX2B and HOXB4 expression (Figure 6h–k). At day 21 of differentiation, RA showed an obvious influence on repressing FOXA2 expression and inducing caudal HOX genes expression in a dose‐dependent manner (Figure S9a–c, Supporting Information). Inhibition of RARα by RO significantly blocked RA‐mediated repression of FOXA2 expression and upregulation of PHOX2B and HOXB4 (Figure 6h–k), indicating that RARα played a dominant role in RA‐mediated FOXA2 repression and neural caudalization. Apart from RARα, we also identified that RARγ was partially involved in RA‐mediated suppression of FOXA2 expression (Figure 6h,i) and RARβ was partially involved in RA‐mediated neural caudalization (Figure 6h,k), respectively. Furthermore, AM580, an agonist of RARα, was proved to mimic the role of RA to suppress FOXA2 expression and promote PHOX2B and HOXB4 expression (Figure 6l,m), confirming the dominant role of RARα in inhibiting FOXA2 expression and promoting caudalization.

### 2.7 Activation of RA and SHH Pathways Promotes the Generation of Caudal SNs

**Figure 7:**
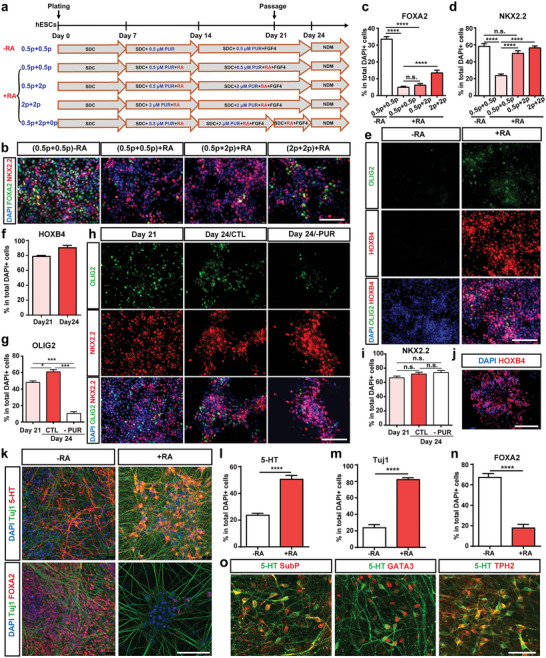
RA promotes caudal SN differentiation. a) Schematic of cell differentiation strategy with RA‐treatment. b–d) Immunofluorescence staining and quantification of FOXA2+ cells (b, c) and NKX2.2+ cells (b, d). e–g) Immunofluorescence staining and quantification of HOXB4+ cells (e, f) and OLIG2+ cells (e, g). h) Immunofluorescence staining of OLIG2 and NKX2.2 at day 24 of differentiation with or without PUR treatment from day 21 to day 24. i) Quantification for NKX2.2+ cells in h. j) Immunofluorescence staining of HOXB4 at day 24 without PUR treatment from day 21 to day 24. k–n) Immunofluorescence staining and quantification of 5‐HT+ cells (k, l), Tuj1+ cells (k, m) and FOXA2+ cells (k, n) at day 42 with or without RA treatment from day 7 to day 21. o) Immunofluorescence staining of SN markers at day 42 with RA treatment. Data are represented as mean ± SEM with three independent experiments. *p < 0.05; ***p < 0.001; ****p < 0.0001; n.s.: no significance. hESCs: human embryonic stem cells; NIM: neural induction medium; SDC: SB431542, DMH1, CHIR99021; PUR: purmorphamine; NDM: neuronal differentiation medium; CTL: control; SubP: substance P. Scale bar: (b, e, h, j, k, o) 100 µm.

Since RA could suppress FP development and promote neural differentiation by inhibiting FOXA2 expression, we further investigated the role of RA in SN differentiation. hPSCs (H9)‐derived NSCs were treated with low concentration of PUR (0.5 µM from day 7 to day 21) with or without RA (**Figure** **7**a). We found that RA decreased the percentage of FOXA2‐positive cells but also unexpectedly decreased the percentage of NKX2.2‐positive cells (Figure 7b–d). In order to obtain more NKX2.2‐positive cells, we changed the concentration of PUR at different time points as shown in Figure 7a. The proportion of FOXA2‐positive cells increased when treated with high concentration of PUR (2 µM) from day 7 to day 21 despite the addition of RA. However, treatment with RA and PUR (0.5 µM for early‐stage and 2 µM for late‐stage) helped to obtain more NKX2.2‐positive SNPs and fewer FOXA2‐positive cells (Figure 7b–d). Thus, the protocol (RA+0.5p+2p) was used for further experiments. Given the role of RA in caudal hindbrain patterning and motor neuron differentiation, we examined the expression of HOXB4 and OLIG2 (a motor neuron progenitor marker) at day 21. The percentage of HOXB4‐positive cells reached ≈80% with a high percentage (≈50%) of OLIG2‐positive cells (Figure 7e–g). It is consistent with the report that co‐activation of RA and SHH signaling pathways could enhance the differentiation of motor neurons.^[^ ^44^, ^45^ ^]^ Given that SHH is required for sustaining OLIG2 expression but not required for sustaining NKX2.2 expression,^[^ ^46^ ^]^ PUR was withdrawn at day 21 to reduce OLIG2 expression. At day 24, the percentage of OLIG2‐positive cells was reduced to <10% while the proportion of NKX2.2‐positive cells maintained at a high level (Figure 7h,i). Besides, withdrawal of PUR did not affect the high percentage of HOXB4‐positive cells at day 24 (Figure 7f,j). Accordingly, we optimized the SN differentiation protocol (RA+0.5p+2p+0p). At day 42, the percentage of the 5‐HT‐positive SNs and Tuj1‐positive neurons was significantly increased, and the proportion of FOXA2‐positive cells was significantly decreased compared to the cells without RA treatment (Figure 7k–n). These SNs expressed not only SN markers (TPH2 and GATA3), but also a caudal SNs marker Substance P (Figure 7o). The optimized SN differentiation protocol could also be applied to two other hPSC lines (H1 and ZSSY001) (Figure S10, Supporting Information). These data indicated that activation of RA and dynamic modulation of SHH signaling pathways facilitated the generation of caudal SNs.

## 3 Conclusion

In conclusion, we found that FOXA2 is not intrinsically required for human SN differentiation. Activation of RA and dynamic modulation of SHH signaling pathways can suppress FP cells differentiation and promote SN differentiation. We exposed for the first time the transcriptomic profiles and subpopulation classification of human SNs at single‐cell level, thus providing valuable information that would fuel the ongoing discovery and innovation in the field of neuroscience research associated with human SNs. This study also provides a new insight into the transcriptional network for human SN development, which would facilitate the development of a more efficient approach to obtain human SNs from hPSCs and may contribute to revealing the underlying pathogenesis of human SN‐related disorders.

## 4 Experimental Section

### Generation of FOXA2‐Lineage‐Tracing hPSC Line

FOXA2‐lineage‐tracing hPSC line was a gift from Prof. Xiaoqing Zhang.^[^ ^12^ ^]^

### Generation of FKO1 hPSC Line

FKO1 hPSC line was generated by inserting stop codons within the exon 2 of FOXA2 gene locus using CRISPR/Cas9‐mediated HDR‐based editing system. Briefly, 5 µg PX459‐sgRNA and 10 µg FOXA2‐Stop‐SV40‐NeoR were transfected to hPSCs using Gene Pulser (Bio‐Rad) in 400‐µL electroporation buffer (KCl 5 mM, MgCl_2_ 25 mM, HEPES 15 mM, Na_2_HPO_4_ 102.94 mM, NaH_2_PO_4_ 47.06 mM [pH 7.2]). 0.4 µg mL^−1^ puromycin was added into medium 24 h after electroporation for 2 days followed by 40 µg mL^−1^ G418 treatment for one week. Drug resistant clones were manually selected and expanded for genomic screening.

### Generation of FKO2 hPSC Line

FKO2 hPSC line was generated by introducing a premature termination codon using CRISPR‐Cas9 technology via NHEJ‐mediated repair pathway.^[^ ^47^ ^]^ Briefly, 5 µg PX459‐sgRNA was transfected to hPSCs as described above. 0.4 µg mL^−1^ puromycin was added into the medium 24 h after electroporation for 2 days and drug resistant clones were picked and expanded for genomic screening.

### Generation of FOXA2‐iOE hPSC Line

FOXA2‐iOE hPSC line was generated on the background of an FKO2 hPSC line by CRISPR/Cas9 mediated gene editing. The coding sequence of human FOXA2 gene was constructed into the iOE plasmid (Addgene, #52 344) to obtain AAVS1‐TRE3G‐FOXA2 vector.^[^ ^48^ ^]^ Then 5 µg PX330‐sgRNA and 10 µg AAVS1‐TRE3G‐FOXA2 were transfected to the FKO2 hPSCs as described above. 0.4 µg mL^−1^ puromycin was added into the medium 24 h after electroporation for one week and drug resistant clones were selected and expanded for genomic screening.

### Generation of FKO‐TPH2EGFP Reporter Cell Line

FKO‐TPH2^EGFP^ reporter cell line was generated on the background of an FKO2 hPSC line by CRISPR/Cas9 mediated gene editing as it was previously described.^[^ ^23^ ^]^

### Differentiation of hPSCs toward SNs

hPSCs were differentiated into SNs with some modifications were previously described.^[^ ^5^ ^]^ Briefly, hPSCs were dissociated by TrypLE into single cells and passaged at the density of 5 × 10^4^ cells cm^−2^ onto Matrigel‐coated plates with mTeSR1 medium and 1 µM Y27632. After 24 h, the medium was changed to NIM (containing DMEM/F12:Neurobasal (1:1), 1 × N2, 1 × B27, 1 × NEAA, 1 × GlutaMAX) with 1.8 µM CHIR, 2 µM SB‐431542 and 2 µM DMH1. At day 7, PUR (0.5 µM, 1 µM or 2 µM) with or without 100 nM RA were added into the medium as indicated. At day 14, 10 ng ml^−1^ FGF4 was added into the medium, PUR with or without RA was added into the medium as indicated. At day 21, cells were dissociated by TrypLE into single cells and reseeded on PO‐Laminin coated coverslips (diameter: 12 mm) at the density of 3 ×10^4^/coverslip. At day 25, NIM was changed to NDM (containing Neurobasal, 1 × N2, 1 × B27, 1 × NEAA, 0.2 mM vitamin C, 2.5 µM DAPT, 10 ng ml^−1^ GDNF, 10 ng ml^−1^ BDNF, 1 ng ml^−1^ TGFβ3, 10 ng ml^−1^ IGF1).

### Quantification and Statistical Analysis

Data were represented as mean ± the standard error of the mean (SEM) with at least three independent biological replicates. Unpaired, two‐tailed student's t‐test was used to compare the difference between two groups. *P<0.05, **P<0.01, ***P<0.001 and ****P<0.0001 were considered to be significant. Data were analyzed using GraphPad PRISM 6.

The sequences for oligos and primers used in this study were listed in Table S4 (Supporting Information). The information for regents and resources used in this study was listed in Table S5 (Supporting Information).

For additional experimental details, please refer to the Supporting Information.

## Conflict of Interest

The authors declare no conflict of interest.

## Author Contributions

T.X. and L.C. contributed equally to this work and are co‐first authors. Conceptualization was done by J.L., T.X., L.C.; formal analysis was done by T.X., L.C.; funding acquisition was done by J.L., L.C.; investigation was done by T.X., L.C.; methodology was done by T.X., L.C., J.D., S.L., M.Z., G.W.; project administration was done by J.L.; resources was done by F.G.; validation was done by Y.L., Z.H.; visualization was done by T.X., L.C; supervision was done by J.L.; writing was done by T.X., L.C., J.L.

## Supporting information

Supporting Information

## Data Availability

The bulk RNA‐sequencing and scRNA‐sequencing data have been deposited in the Gene Expression Omnibus (GEO) dataset under the accession number (GSE232830). The following secure token has been created to allow review of record GSE232830 while it remains in private status: ynilomcutngxlex.
