# Supplementary material for: Uncovering the role of FOXA2 in the Development of Human Serotonin Neurons
Source: Adv Sci (Weinh). 2023 Sep 7;10(32):2303884. doi: 10.1002/advs.202303884 (PMC10646255; doi:10.1002/advs.202303884)
Supplement: Supplementary file 1 — Supporting Information [file ADVS-10-2303884-s001.pdf]

ADVANCED SCIENCE
Open Access

Supporting Information

for *Adv. Sci.*, DOI 10.1002/advs.202303884

Uncovering the role of FOXA2 in the Development of Human Serotonin Neurons

*Ting Xu, Lining Cao, Jinjin Duan, Yingqi Li, You Li, Zhangsen Hu, Shuanqing Li, Meihui Zhang, Guanhao Wang, Fei Guo and Jianfeng Lu**

Supporting Information

Uncovering the role of FOXA2

in the development of human serotonin neurons

*Ting Xu, Lining Cao, Jinjin Duan, Yingqi Li, You Li, Zhangsen Hu, Shuanqing Li, Meihui Zhang, Guanhao Wang, Fei Guo, Jianfeng Lu**

Experimental Section

hPSC lines and cell culture

Three hPSC lines (2 hESC lines-H9, H1 and 1 iPSC line-ZSSY001) were used in this study. All hPSC lines were cultured in mTeSR1 medium (Stem Cell Technologies, Cat. 05850) on Matrigel in the incubator (37°C, 5% CO_2) and passaged with TrypLE. All genetically engineered hPSC lines retained pluripotency and had normal karyotypes.

Plasmid design and construction

To construct the FKO1 or FKO2 targeting vectors, the oligos of single guided RNA (sgRNA) were annealed and cloned into PX459 backbone plasmid to form PX459-sgRNA. To construct the FOXA2-iOE targeting vector, the oligos of single guided RNA (sgRNA) were annealed and cloned into PX330 backbone plasmid to form PX459-sgRNA.

For FKO1 donor plasmid (FOXA2-Stop-SV40-Neo) construction, three stop codons in different reading frames (5'-TGAGTAGGTAG-3') and SV40-Neo (a hybrid gene consisting of SV40 promoter driving the Neomycin resistance gene) were sequentially assembled into pUC19 plasmid to get pUC19-Stop-SV40-Neo. Then the left and right homology arms flanking the target site were assembled into the pUC19-Stop-SV40-Neo to form the donor vector. For FOXA2-iOE donor plasmid construction, the FOXA2 coding sequence were amplified from the pENTER-FOXA2 plasmid (Vigene Biosciences, Shandong, China) and cloned into AAVS1-TRE3G-EGFP to replace the EGFP. The sequences of sgRNA oligos are listed in **Table S4**.

Single-cell RNA-seq library preparation and sequencing

$TPH2^{EGFP}$ reporter cell lines were used for this assay. At day 42 of differentiation, cells were dissociated by accutase and filtered through cell strainer (40 μm) to obtain single cells. Highly purified EGFP-labeled SNs were then derived by fluorescence-activated cell sorting (FACS) as we previously described.[1]

Single-cell suspension was loaded to the 10x Chromium to capture 8000-10000 single cells according to the instructions for the 10X Genomics Chromium Single-Cell 3' kit (V3). cDNA amplification and library construction were performed according to standard protocols. Sequencing was carried out on an Illumina NovaSeq 6000 platform using 150

bp double-end reads, with a minimum depth of 20,000 reads per cell. The output CellRanger expression profile matrix was loaded into Seurat (version 4.1.0) to filter out low quality cells from scRNA-seq data. The filtered data was downscaled and clustered. Quality control involved removing low-quality cells with >25% reads mapping to mitochondria or that expressed fewer than 500 genes per cell. Then the Doublet Finder R package was used to filter for the second time to remove the doublets.

The Illumina sequencing results were converted to FASTQ format using bcl2fastq software (version 5.0.1). The scRNA-seq sequencing data were compared to reference genome using CellRanger software. This analysis enabled the identification and quantification of cellular and individual cellular 3' end transcripts in the sequenced samples. (https://support.10xgenomics.com/single-cell-gene expression/software/pipelines/latest/what-is cell-ranger, version 7.0.0)

Standard procedures for dimensionality reduction and clustering were performed using the Seurat package (version 4.1.0). For visualization, cells were projected into 2-dementional space using the Uniform Manifold Approximation and Projection (UMAP). Cellranger aggr was used for data normalization and the integration of the datasets from the two samples, while Harmony was used to remove the batch effects. The downstream clustering was then performed. Briefly, gene expression values were calculated using the LogNormalize method on Seurat system. PCA was performed using the normalized expression values, top 20 PCs were used for clustering and Findcluster analysis. The marker genes of each cluster were analyzed based on Findallmarker (selection criteria: expressed in more than 10% of cells in each cluster, P value ≤ 0.01, gene expression logFC ≥0.26). Hypergeometric testing was used to perform KEGG enrichment analysis on the differential genes within each cluster, relative to other clusters obtained from FindAllMarkers analysis.

Comparison of scRNA datasets of *in vitro* human SNs with *in vivo* mouse SNs

For cross-species comparison analysis of scRNA-seq data, the two scRNA-seq datasets of WT mouse SNs reported by Okaty et al. were downloaded from the Gene Expression Omnibus (GEO) repository (GSM4303413, GSM4303412).[2] The orthologous genes across species (mouse-WT1, mouse-WT2, human-WT, human-FKO SNs) were identified

by BLAST, and only the one-to-one orthologous genes (n=57612) were included for subsequent analysis, the genes without one-to-one matches or with one-to-many matches were excluded. Cross-species integration of the scRNA-seq datasets and batch correction were performed using the LIGER (Linked Inference of Genomic Experimental Relationships) algorithm, which was reported to be an excellent algorithm for cross-species integration of scRNA-seq datasets.[3] One of the scRNA datasets of WT mouse SNs is a biological replicate of the other dataset,[2] we thus combined the two mouse scRNA-seq datasets into one dataset.

Immunofluorescence staining of cells

Cells seeded on the coverslips were fixed with 4% paraformaldehyde for 30 minutes and washed with DPBS for three times at room temperature (RT). Then cells were incubated with blocking buffer (10% donkey serum and 0.2% Triton-X100 in DPBS) for 30 minutes at RT, followed by incubation with primary antibodies (diluted in DPBS, containing 5% donkey serum and 0.2% TritonX-100) at 4°C overnight. Cells were then washed with DPBS for three times, followed by 45 minutes of incubation with fluorescent secondary antibodies and DAPI (diluted in DPBS, containing 5% donkey serum) at RT. Then cells were washed with DPBS and mounted onto slides with anti-fade Fluoromount-G solution. The information for antibodies used in this study is listed in **Table S5**.

mRNA extraction and qPCR

Total RNA was extracted using RNA extraction kit (Magen, China) and diluted in RNAase free water. 1 μg of RNA was reverse transcribed to cDNA using PrimeScript^{TM}RT reagent kit (TAKARA, Japan). qPCR was performed using the SYBR Green Mix (Bio-Rad, USA) in a BioRad CFX96 Thermal Cycler (Bio-Rad, USA). Housekeeping gene *GAPDH* was used to normalize mRNA levels between different samples. Primer sequences are listed in **Table S4**.

Western blotting

Cells were lysed in RIPA buffer supplemented with proteinase inhibitors cocktails and PMSF on ice for 10 minutes. Protein quantification was performed using BCA kit, followed by denaturation of protein in sample loading buffer at 100°C for 10 minutes. 30 μg of total protein was loaded and separated in 10% SDS-PAGE gel using running buffer. Then

proteins were transferred to PVDF membrane in transfer buffer using semi-dry transfer device (Bio-Rad, USA), followed by blocking in TBST containing 5% non-fat milk for 1 hour at RT. After washing with TBST for three times, the membrane was incubated in primary antibodies diluted in TBST containing 5% non-fat milk at 4°C overnight on the shaker. After washing with TBST for three times, the membranes were incubated in HRP-conjugated secondary antibodies at RT for 1 hour. After washing for three times, the membranes were incubated in ECL working solution (Bio-Rad, USA) for 1 minutes and transferred to the Amersham Imager 600 (GE, USA) immediately to detect the signals. The information for antibodies used in this study is listed in **Table S5**.

Conditioned medium assay

Collection of conditioned medium and incubation assay were performed as previously described with some modifications.[4] Briefly, at day 21 of differentiation, medium was removed and the cells were washed in DPBS for three times. The fresh NIM without patterning cues was added into the cultures and collected after 3 days. The conditioned medium was filtered and mixed with fresh NIM at 1:2 ratio and then incubated with cells at day 7 of differentiation for one week. The medium was replaced by fresh conditioned medium every other day. At day 14 of differentiation, the cells were fixed and detected for NKX6.1 expression.

Measurement of 5-HT release by ELISA

At day 52 of differentiation, fresh NDM was added to SN culture and incubated at 37°C for 1 hour (with/without 10 μM EO) or 20 min (with/without 56 mM KCl). All the supernatants were collected and the extracellular 5-HT level was measured using ELISA kit according to the manufacturer's instructions. Whole-cell slides were scanned with Pannoramic MIDI (3DHISTECH, Budapest, Hungary) and 5-HT-positive SNs per slide were counted with ImageJ software. Then the concentrations of 5-HT released by each SN was calculated as 5-HT concentration divided by the number of SNs per slide.

Electrophysiological recording

Whole cell electrophysiology recording was performed on cells at day 42 of differentiation as we previously described. [1, 5] Briefly, coverslips with $TPH2^{EGFP}$ reporter SNs were carefully transferred to the recording chamber and perfused with balanced salt solution.

SNs were identified by EGFP signals under the fluorescence microscope. The recording electrodes were filled with an internal fluid (20 mM KCl, 10 mM Na^+-HEPES, 121 mM K^+-gluconate, 10 mM BAPTA, 4 mM Mg^{2+}-ATP, pH = 7.2) and the electrical resistance was 3~5 MΩ. To detect activities of Na^+/K^+ channel, Na^+/K^+ currents were triggered by stepped voltages from -40 mV to +30 mV in 5 mV increments and recorded. Then, the currents (2 pA increment, from -14 pA to +14 pA) were injected to the SNs to evoke the APs. Spontaneous APs and sEPSC were recorded in current-clamp mode (0 mA) and voltage-clamp mode (-60 mV), respectively. Data were acquired by Clampex 10.2 software through DigiData-1440A converter and analyzed by Clampfit 10.2, Origin and GraphPad Prism software.

Inhibition/activation assays for RA receptors

For RAR inhibitory assay: at day 7 of differentiation, 0.5 μM purmorphamine with 100 nM RA were added into the medium. Then the selective antagonists for the three RA receptors (2 μM of RO 41-5253: inhibitor for RARα; 1 μM of LE135: inhibitor for RARβ; 1 μM of LY 2955303: inhibitor for RARγ) were treated to the cells for one week, respectively. For RARα activation assay: at day 7 of differentiation, 0.5 μM purmorphamine was added into the medium. Then 100 nM of AM580, a selective agonist of RARα, was added to medium for one week. At day14 of differentiation, the cells were investigated by immunofluorescence assay.

RNA sequencing

Total RNA was extracted from different groups with three replicates at day 14 of differentiation. RNA-seq transcriptome library was prepared using TruSeq™ RNA sample preparation Kit from Illumina (San Diego, CA) with 1 μg of total RNA. Then the paired-end RNA-seq library was sequenced with the Illumina HiSeq xten/NovaSeq 6000 sequencer (2 × 150 bp read length) (Majorbio, China). The raw paired reads were trimmed and the quality was controlled by SeqPre (https://github.com/jstjohn/SeqPrep) and Sickle (https://github.com/najoshi/sickle), followed by aligning to the reference genomic using HISAT2. More than 6.77Gb clean data was obtained for each sample. DEGs analysis was performed by DESeq2 with adjusted-P value <=0.05. GO functional enrichment assay and KEGG pathway analysis were performed using Goatools and KOBAS.

Supplemental Figure

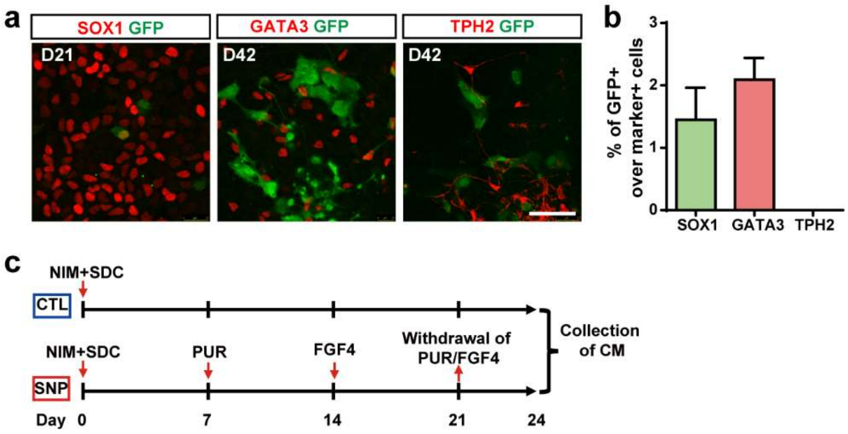

Figure S1. Investigation of the identity of FOXA2+ cells. (**a**) Immunofluorescence staining of SOX1, GATA3 and TPH2 at key stages of differentiation (day 21 and 42) of FOXA2-lineage-tracing hESCs towards SNs. (**b**) Quantification for **a**. n = 3 independent experiments. (**c**) Schematic diagram for collecting conditioned medium from control and SNP with PUR treatment. NIM: neural induction medium; SDC: SB431542, DMH1, CHIR99021; PUR: purmorphamine; NDM: neuronal differentiation medium; CTL: control; CM: conditioned medium; SNP: serotonergic progenitors. Scale bar: (**a**) 50 μm.

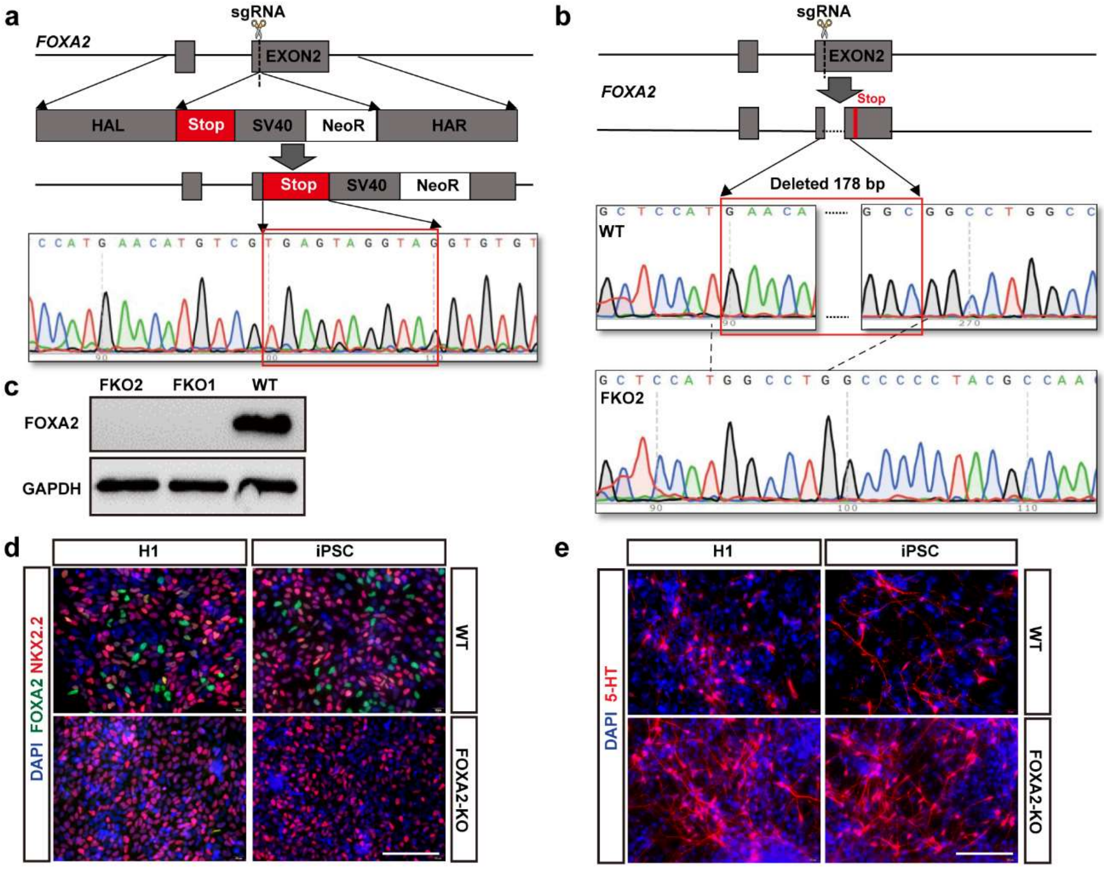

Figure S2. Generation of FKO hPSC lines and construction of TPH2^{EGFP} reporter cell line on the background of FKO2 cells. (**a, b**) Schematic diagram for the 1st FKO (**a**) and the 2nd FKO (**b**) strategies and Sanger sequencing chromatogram for FKO cell lines. (**c**) Western blotting of FOXA2 for three cell lines (WT-H9, FKO1-H9 and FKO2-H9) at day 14 of differentiation towards SNs. (**d**, **e**) Immunofluorescence staining of FOXA2 and NKX2.2 at day 21 (**d**) and 5-HT at day 42 (**e**) of differentiation towards SNs from H1 and iPSC-derived WT or FKO hPSCs. n = 3 independent experiments. Scale bar: (**d**, **e**) 100 µm.

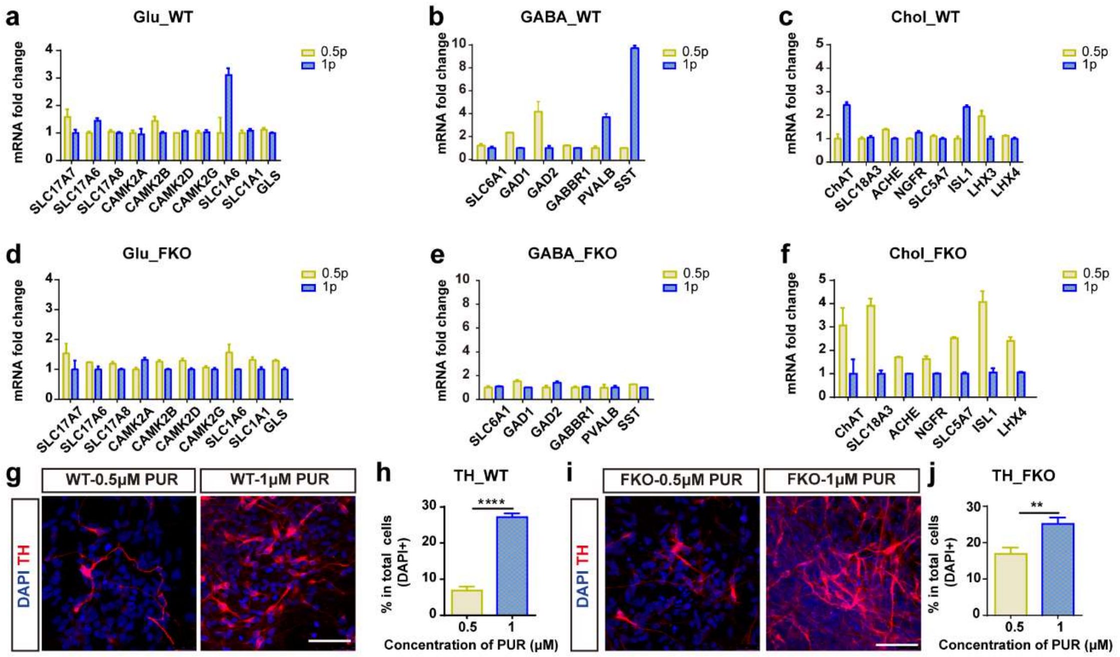

Figure S3. Evaluation of the influence of PUR on the proportion of other neuron types in the differentiation medium of WT and FKO cells. (**a-c**) mRNA expression levels of glutamatergic (**a**), GABAergic (**b**) and cholinergic (**c**) markers in WT cells treated with different concentrations of PUR. (**d-f**) mRNA expression levels of glutamatergic (**d**), GABAergic (**e**) and cholinergic (**f**) markers in FKO cells treated with different concentrations of PUR. (**g, h**) Immunofluorescence staining (**g**) and quantification (**h**) of TH+ cells in WT cells treated with different concentrations of PUR. (**i, j**) Immunofluorescence staining (**i**) and quantification (**j**) of TH+ cells in FKO cells treated with different concentrations of PUR. n = 3 independent experiments. **p<0.01; ****p<0.0001. Scale bar: (**g, i**) 100 μm.

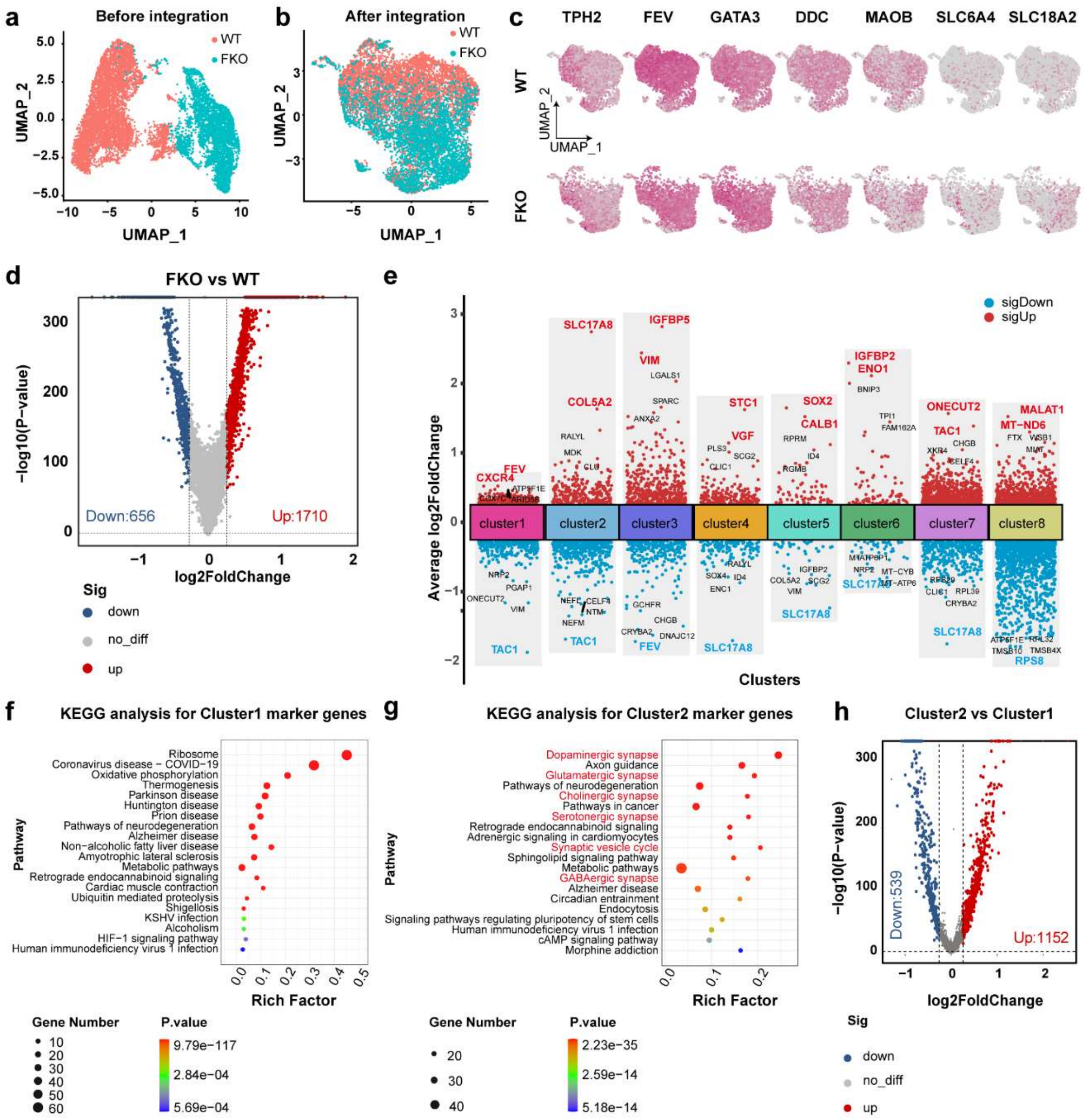

Figure S4. Comparative analysis of subpopulation composition and transcriptomic profiles of WT- and FKO-SNs. (**a**) UMAP plot of the datasets before integration. (**b**) UMAP plot of the datasets after integration. (**c**) Expression patterns of the key serotonergic marker genes across individual serotonin neurons presented as UMAP plots after integration. (**d**) The volcano plots of the upregulated and the downregulated DEGs in FKO-SNs compared to WT-SNs. (**e**) The volcano plots of the top five upregulated- and downregulated-genes in each cluster for the combined dataset. (**f**) KEGG enrichment analysis of the cluster 1 marker genes. (**g**) KEGG enrichment analysis of the cluster 2 marker genes. (**h**) The volcano plots of the upregulated and the downregulated DEGs in cluster 2 compared to cluster 1.

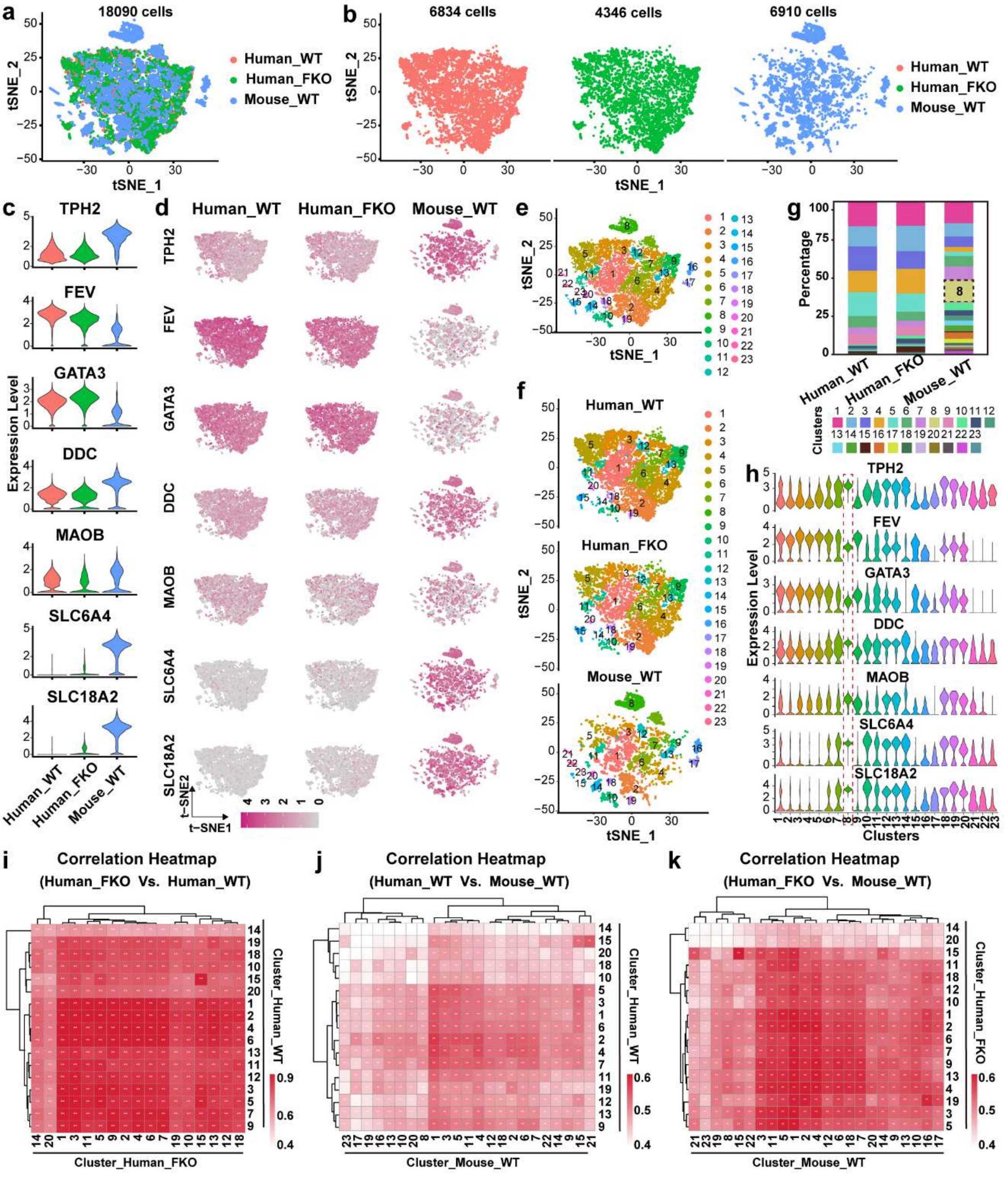

Figure S5. Cross-species integration and comparison of scRNA-seq datasets of human_WT, human_FKO and mouse_WT *TPH2*+ SNs. (**a-b**) Combined (**a**) and split (**b**) tSNE plot showing the distribution of individual *TPH2+* SNs of human_WT, human_FKO and mouse_WT groups. (**c**) Violin plot showing the expression level of serotonergic markers in human_WT, human_FKO and mouse_WT *TPH2*+ SNs. (**d**) tSNE plot showing the expression pattern of serotonergic markers by human_WT, human_FKO and mouse_WT *TPH2*+ SNs at single cell level. (**e-f**) Combined (**e**) and split (**f**) tSNE plot

illustrating the distribution of the 23 clusters of human_WT, human_FKO and mouse_WT *TPH2*+ SNs. (**g**) The bar graph showing the percentage of each cluster in human_WT, human_FKO and mouse_WT *TPH2*+ SNs. (**h**) Violin plot showing the expression of SN markers in the 23 clusters from the combined datasets of human and mouse *TPH2*+ SNs. (**i**) Heatmap showing the Spearman correlation of the clusters between human_FKO and human_WT SNs. (**j**) Heatmap showing the Spearman correlation of the clusters between human_WT and mouse_WT SNs. (**k**) Heatmap showing the Spearman correlation of the clusters between human_FKO and mouse_WT SNs. The correlation coefficient is represented by the color bar: a higher coefficient indicates a stronger correlation.

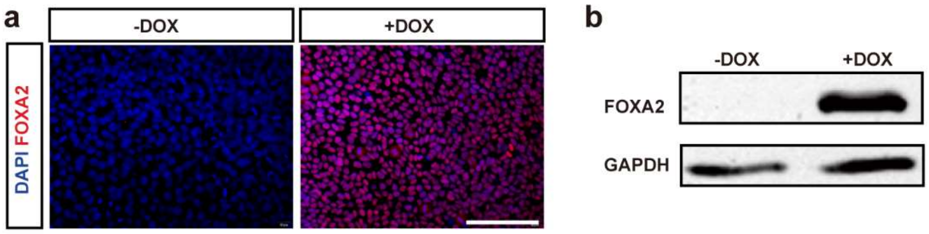

Figure S6. Validation of FOXA2-iOE hPSC line (H9). (**a-b**) Immunofluorescence staining (**a**) and western blotting (**b**) of FOXA2 in WT and FOXA2-iOE hPSCs after DOX treatment for 3 days. WT: wide type; DOX: doxycycline. Scale bars: (**a**) 100 μm.

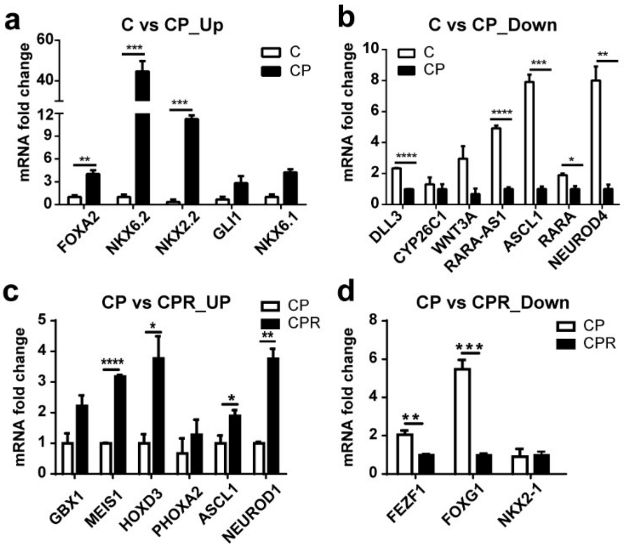

Figure S7. Verification of DEGs by qPCR. (**a, b**) mRNA expression levels of the upregulated (**a**) and the downregulated (**b**) DEGs in WT hPSCs-derived cells at day 14 of differentiation with or without PUR treatment. (**c, d**) mRNA expression levels of the upregulated (**c**) and the downregulated (**d**) DEGs in WT hPSCs-derived cells at day 14 of differentiation with or without RA treatment. *p<0.05; **p<0.01; ***p<0.001; ****p<0.0001. C: treatment with SDC; CP: treatment with SDC and PUR; CPR: treatment with SDC, PUR and RA.

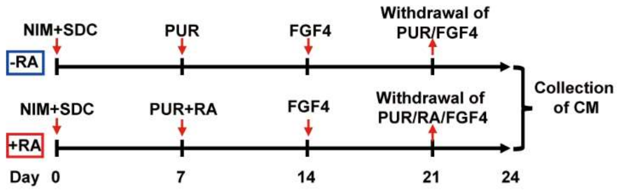

Figure S8. Schematic diagram for the collection of conditioned medium. Schematic diagram for conditioned medium collection from the differentiated cells with PUR treatment and PUR plus RA treatment. NIM: neural induction medium; SDC: SB431542, DMH1, CHIR99021; PUR: purmorphamine; NDM: neuronal differentiation medium; CTL: control; CM: conditioned medium; SNP: serotoninergic progenitors.

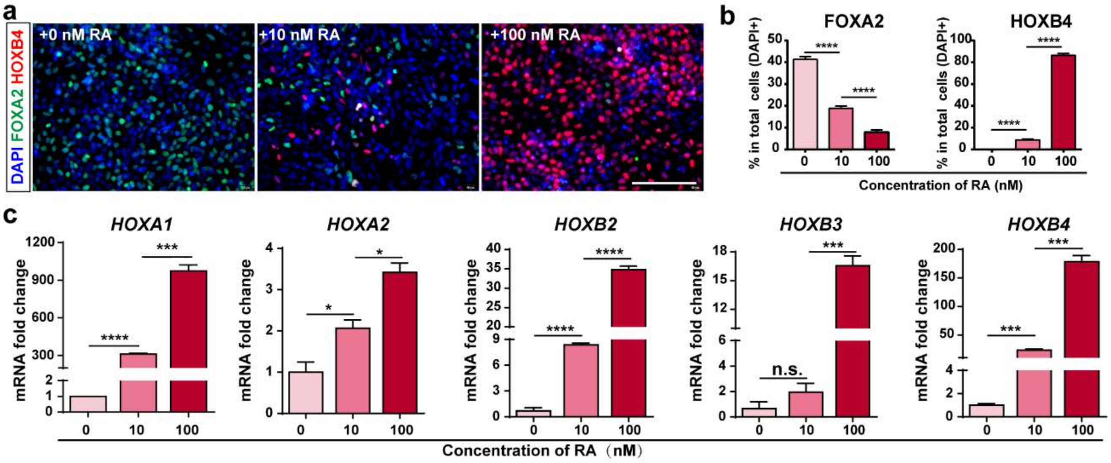

Figure S9. Evaluation of the influence of RA on FOXA2 expression and neural caudalization. Immunofluorescence staining (**a**) and quantification (**b**) of FOXA2+ cells or HOXB4+ cells in response to increasing concentrations of RA. (**c**) mRNA expression levels of caudal HOX genes in response to increasing concentrations of RA. *p<0.05; ***p<0.001; ****p<0.0001; n.s.: no significance. Scale bars: (**a**) 100 µm.

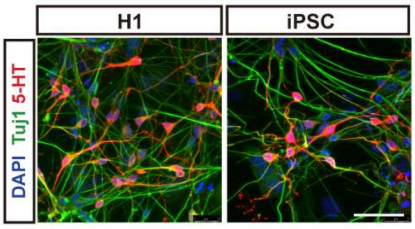

Figure S10. Differentiation of caudal SNs from hPSCs. Immunofluorescence staining of 5-HT and Tuj1 at day 42 of differentiation from H1 and ZSSY001 (an iPSC line) towards caudal SNs. Scare bar = 50 µm.

Supplemental Table

Table S1. The number of total cells and serotonergic marker positive cells

Group	Number and percentage of marker positive cells							
	Total	*TPH2*	*FEV*	*GATA3*	*DDC*	*MAOB*	*SLC6A4*	*SLC18A2*
WT	6000	6000/ 100%	5913/ 98.6%	5796/ 96.6%	5368/ 89.5%	4408/ 73.5%	1205/ 20.1%	1198/ 20.0%
FKO	3891	3891/ 100%	3724/ 95.7%	3768/ 96.8%	3047/ 78.3%	1997/ 51.3%	1005/ 25.8%	1357/ 34.9%

Table S2. List of the expression levels of interested genes in the clusters of WT- and FKO-SNs. (The numbers represent relative expression levels of genes calculated by the AverageExpression function in Seurat.)

GeneName	TPH2	GATA3	LMX1B	SLC18A2	SLC29A4	HTT	MAOA
WT_Cluster1	3.37	7.86	0.38	0.23	0.20	0.25	0.09
FKO_Cluster1	4.69	11.90	0.83	0.49	0.31	0.51	0.32
WT_Cluster2	2.33	6.31	0.41	0.25	0.18	0.35	0.14
FKO_Cluster2	2.73	9.24	0.84	0.67	0.30	0.52	0.62
WT_Cluster3	1.87	5.13	0.32	0.21	0.17	0.27	0.08
FKO_Cluster3	2.31	6.75	0.64	0.75	0.27	0.50	0.30
WT_Cluster4	4.92	7.27	0.41	0.15	0.15	0.27	0.06
FKO_Cluster4	5.14	9.26	1.15	0.28	0.31	0.36	0.39
WT_Cluster5	2.27	6.73	0.35	0.15	0.18	0.28	0.09
FKO_Cluster5	2.56	9.99	0.71	0.43	0.27	0.54	0.34
WT_Cluster6	5.61	3.69	0.40	0.35	0.19	0.33	0.10
FKO_Cluster6	5.86	7.08	0.70	1.09	0.44	0.67	0.37
WT_Cluster7	2.59	7.25	0.44	0.34	0.19	0.23	0.09
FKO_Cluster7	3.06	9.73	0.85	0.79	0.40	0.69	0.53
WT_Cluster8	1.00	1.27	0.14	0.03	0.15	0.20	0.09
FKO_Cluster8	1.76	3.97	0.25	0.20	0.29	0.49	0.16

Table S3. List of the selected SN diversity related genes from the published studies.

**The genes listed below were reported from the article by Okaty et al., 2015.[6]**							
F2RL2	CALCR	EDNRB	GPR101	RAMP1	GRM4	GRIK3	PTGER3
OPRK1	HTR2A	GALR1	RXFP3	CXCR4	ADRB1	GLP1R	ADRA1A
GPR139	HTR2C	OPRM1	HTR1D	HTR1B	NPY2R	ADGRL2	ADGRG2
HTR5B	HCRTR2	CRHR2	PKD2L1	LPAR1	NPY1R	CHRM1	OXTR
P2RY1	GRM7	GPR35	CYSLTR1	CHRM2	NTSR1	CNR1	GPR22
GRM5	TACR3	CCKAR	PTH1R	SSTR1	RXFP1	GRM8	NMBR
PRLHR	PTH2R	GABRA4	GLRA2	CFTR	SYT17	CACNA1I	GRIK1
IL1RAPL1	CHRNA4	CACNA1G	HCN4	SLC6A1	TRPM3	TRPC5	KCNIP4
KCNT2	RYR3	KCNV1	PIEZO2	CHRNB4	KCNH8	NOS1	KCNAB1
KCND3	DPP10	CACNA1E	CACNG3	ORAI1	KCNA5	SLC5A7	FXYD5
CLIC1	CNIH3	KCNIP1	SYTL5	KCNQ5	GRIA3	GLRA3	ITPR2
KCNS3	SV2C	SLC32A1	GAD2	CACNG5	PLAGL1	MEIS2	ONECUT2
NPAS1	SOX5	BCL11A	NKX2-2	NR2F2	HOXA2	POU3F1	TCF4
RARA	ZFP560	CBFA2T3	SLC44A2	NPAS4	RORB	FOXP1	SOX1
KIT	PBX1	PBX3	ZFP467	ZFPM1	ESR2	EN1	DAP
FOXA1	PAX5	EN2	SOX14	NLRX1	TNFRSF8	CREB3L1	IRAK3
CREB3L2	ETV5	MEIS1	NKX6-1	CREM	ZFPM2	TRIM15	BHLHE22
HOXD3	ELK3	TRIM25	HOXB2	NR2F1	CNTN6	CDH9	PLXNB1
PCDH18	PTPRZ1	PCDH10	CDH10	TENM2	SEMA3E	COL19A1	SEMA5A
DSCAM	CHL1	SLIT2	NRXN1	PCDHB16	KIRREL	CDH8	PCDH19
NEGR1	CDH2	PCDH8	EPHA4	THY1	CNTN3	RELN	CDH6
ITGB1	SEMA4D	EMILIN2	COL8A1	CDH13	COL14A1	ROBO1	RET
SPOCK1	ROBO2	PRKCA	CDH11	CDH4	PARVA	CNTN5	CDH23
NRXN3							
**The genes listed below were reported from the article by Okaty et al., 2020.[2]**							
GABBR1	ADRA1B	ADRA2C	HTR1A	HCRTR1	HRH1	GRM1	ADRA2A
HRH3	GPR149	PROKR2	GPR88	S1PR3	PTGER4	GABBR2	HTR7
SSTR2	TACR1	TPH2	SLC18A2	CHGB	CHGA	TRH	PDYN
GAL	GHRH	ADCYAP1	CRH	NMB	NPB	PENK	SST
SCN9A	KCNH7	KCNQ3	KCNC4	CACNG4	SCN7A	KCNG4	KCNN3
KCNJ4	KCNC1	HCN1	KCNB2	CACHD1	KCNC2	CACNA2D3	KCNF1
KCNA1	KCNJ12	KCND2	KCNA4	TRPC3	KCTD8	KCNK2	KCTD4
KCNH5	GABRQ	GABRE	GRIA1	GRIN3A	GABRG3	GRIN2A	GABRA2
GABRG2	GABRA1	GABRA5	S100A10	S100A11	S100A16	CALB1	NECAB2
NECAB1	CALB2	RYR2	S100B	HS3ST4	HS3ST5	NDST3	HS3ST1

EXT1	NDST4	HS3ST6	SULF2	HS3ST2	HS3ST3A1	SULF1	
**The genes listed below were reported from the article by Okaty et al., 2019.**[7]							
FEV	GATA2	GATA3	LMX1B	DDC	MAOA	MAOB	TAC1
CARTPT	GAD1	SLC17A8	MET	DRD2			
**The genes listed below were reported from the article by Ren et al., 2019.**[8]							
ADM	PTHLH	RLN1	EDN3	BCL11B	NPAS2	E2F1	EGR1
EDN3	BCL11B	NPAS2	E2F1	EGR1	SOX4	ZFP521	TOX
RXRG	POU2F2	ZFP503	IRX2	TSHZ1	ARNTL	ZBTB7C	TSHZ2
MBD4	ARID5B	TOX3	SOX13	ZEB2	SCX	MYC	NPAS3
NR4A1	FOXO1	MAFB	TSHZ3	MYB	MEF2C	ESR1	MAF
SATB1	AFF2	POU3F2	NFIB	NFIX	ZFP536	SATB2	TFEB
POU3F3	ZFP599	KLF5	POU6F2	CHRNA3	ADRB2	GIPR	PROKR1
HTR1F	HTR3A	HTR4	HTR5A	HTR6			
**The genes listed below were reported from the article by Huang et al., 2019.**[9]							
MAX	UNCX	ZFP46	NR3C1	PTMA	CITED2	KLF6	ZCCHC18
NR3C1	PTMA	CITED2	KLF6	ZCCHC18	ZBTB20	UNC5C	TENM3
RTN4RL1	TSPAN7	SEMA6A	SHTN1	TMTC1	NXPH4	SDK2	CLSTN2
EPB41L4B	NXPH1	FAT3	PLXNA4	NRCAM	CNTNAP2	NTM	PCDH7
PCDH11X	LSAMP	PCDH15	CBLN4	EPHA5	CBLN2	CD47	AMIGO2
CADM1	NRP2	CNTN1	PTPRT	PTPRM	LTBR	IGSF1	OSBPL1A
FGFR1	GFRA1	GRIA2	KCNAB2	CACNA2D1	CACNA2D2	SLC6A17	SYNGR3
SLC6A4	QDPR	SLC22A3	SLC24A3	SULT4A1	SLC36A1	SYT13	

Table S4. List of oligos and primers used in this study.

Item	Orientation	Sequence (5'-3')
**sgRNA oligos**		
Targeting *FOXA2* gene		ATGAACATGTCGTCGTACGT
Targeting *AAVS1* locus		GGGGCCACTAGGGACAGGAT
Targeting *TPH2* gene		ATATCTGGGGATTTGATGCC
**PCR primers**		
Verification for FOXA2 knockout	forward	CTCCGTGAGCAACATGAACG
	reverse	TCGTTGAAGGAGAGCGAGTG
Verification for TPH2-EGFP reporter: 5TP	forward	AGACCTGGACTAAAGCCCCA
	reverse	TCGACGTCACCGCATGTTAG
Verification for TPH2-EGFP reporter: 3TP	forward	CAACCTCCCCTTCTACGAGC
	reverse	TTGATCTCTCCCTGATGTGTCT
Verification for TPH2-EGFP reporter: TN	forward	CCCTCGTACCAATGAGGGTT
	reverse	AGATGCAGTTTGGTTAAGGACA
**qPCR primers**		
GAPDH	forward	TCAAGATCATCAGCAATGCC
	reverse	CGATACCAAAGTTGTCATGGA
SHH	forward	AGCGATTTAAGGAACTCACC
	reverse	CTTACACCTCTGAGTCATCAG
NTN1	forward	CTGCATAAAGATCCCTGTAGC
	reverse	CTTGCAGTAGGAATCGCAG
SPON1	forward	CAGTTCCCAGGTTGTAGGA
	reverse	ACGTTCCTGAATTCCACCT
NESTIN	forward	GAAGGGCAATCACAACAGGTG
	reverse	GGGGCCACATCATCTTCCA
NCAD	forward	ATTTTTCCCTCGACACCCGAT
	reverse	TCCCAGGCGTAGACCAAGA
FOXA2	forward	GAGTTAAAGTATGCTGGGAGC
	reverse	GTTCATGTTGCTCACGGAG
PHOX2B	forward	GCAGATAACAAATTTCCTCGGT
	reverse	GTGAAGAGTTTGTAAGGAAACCC
RARA	forward	AAGCCCGAGTGCTCTGAGA
	reverse	TTCGTAGTGTATTTGCCCAGC
RARB	forward	TCCGAAAAGCTCACCAGGAAA
	reverse	GGCCAGTTCACTGAATTTGTCC
RARG	forward	ATGCTGCGTATCTGCACAAG
	reverse	AGGCAAAGACAAGGTCTGTGA

NKX6.2	forward	GAGCAGACCAAGTACCTGG
	reverse	TCTGGAACCAGACCTTCAC
NKX2.2	forward	CCTTCAGTACTCCCTGCAC
	reverse	TGTCATTGTCCGGTGACTC
NKX6.1	forward	GAGTCAGGTCAAGGTCTGG
	reverse	CTCTTCCTCGTTCTCCGAG
GLI1	forward	AGCGTGAGCCTGAATCTGTG
	reverse	CAGCATGTACTGGGCTTTGAA
DLL3	forward	CGTCCGTAGATTGGAATCGCC
	reverse	TCCCGAGCGTAGATGGAAGG
CYP26C1	forward	GAAACGCTGCACTGGTTAGTT
	reverse	CAGCAGGTGCGTCTTGAAC
WNT3A	forward	AGATTGGCATCCAGGAGTG
	reverse	CTCCCTGGTAGCTTTGTCC
RARA-AS1	forward	TTATCCTCACAGCAACTCCA
	reverse	CATAGCCTTGCTGAGACCT
ASCL1	forward	CCCAAGCAAGTCAAGCGACA
	reverse	AAGCCGCTGAAGTTGAGCC
NEUROD4	forward	GAGAGCTAGTCAACACACCATC
	reverse	GCATCCCATAAGTACCTGGTCTG
GBX1	forward	GCCCGTAAGAAACCCCAAGAT
	reverse	CTGCTCCATTTGTTGGTGCTG
MEIS1	forward	GGGCATGGATGGAGTAGGC
	reverse	GGGTACTGATGCGAGTGCAG
HOXD3	forward	CGGCAACTTCGTCGAGTCC
	reverse	ATGAGGGTCGCAAGGTCCA
PHOX2A	forward	GTGCCCTACAAGTTCTTCC
	reverse	CTCACGCGTGTAAATGTCG
NEUROD1	forward	GTCTCCTTCGTTCAGACGCTT
	reverse	AAAGTCCGAGGATTGAGTTGC
FEZF1	forward	ATGGACAGTAGCTGCCACAAC
	reverse	TTTGGACGTGCTCATCATGTT
FOXG1	forward	CCGCACCCGTCAATGACTT
	reverse	CCGTCGTAAAACTTGGCAAAG
LMX1B	forward	TTCCTGATGCGAGTCAACGAG
	reverse	GCAGTACAGTTTCCGATCCCG
NKX2.1	forward	AGGACACCATGAGGAACAG
	reverse	CATGTTCTTGCTCACGTCC

HOXA1	forward	GGGTGTCCTACTCCCACTCA
	reverse	GGACCATGGGAGATGAGAGA
HOXA2	forward	CGTCGCTCGCTGAGTGCCTG
	reverse	TGTCGAGTGTGAAAGCGTCGAGG
HOXB2	forward	CCTAGCCTACAGGGTTCTCTC
	reverse	CACAGAGCGTACTGGTGAAAAA
HOXB3	forward	AACGCCTTACACTCCATGACC
	reverse	ATTCTGGTGGGCTTTACCGAA
HOXB4	forward	AAAGAGCCCGTCGTCTACC
	reverse	GTGTAGGCGGTCCGAGAG
SLC17A7	forward	CTGGGGCTACATTGTCACTCA
	reverse	GCAAAGCCGAAAACTCTGTTG
SLC17A6	forward	TGGACATGGTCAACAACAGCA
	reverse	GGAACCGTGGATCATCCCC
SLC17A8	forward	CCTCCCCAAGCGTTACATCAT
	reverse	GCTGTCTGAATTTCCGGTTTTCC
CAMK2A	forward	ACCACTACCTGATCTTCGACC
	reverse	CCGCCTCACTGTAATACTCCC
CAMK2B	forward	CTCTACGAGGATATTGGCAAGGG
	reverse	GCTTCTGGTGATCTCTGGCTG
CAMK2D	forward	AGTCAGAAGAGACTCGTGTGT
	reverse	TGATGGGTACTGTTGGTGACC
CAMK2G	forward	ACGTGAGGCTCGGATATGTC
	reverse	ACGAGGTAGTGAAACCCTTCT
SLC1A6	forward	CTCAACCTGGGTCAGATCACA
	reverse	CCGACCGACGTAAGCACAA
SLC1A1	forward	TTCTAATGCGGATGCTGAAACT
	reverse	CGCGCAGACCAATTTTTCC
GLS	forward	AGGGTCTGTTACCTAGCTTGG
	reverse	ACGTTCGCAATCCTGTAGATTT
SLC6A1	forward	GGGTATGGAAGCTGGCTCCTA
	reverse	AGGGGTTGTCGCACTGTTTC
GAD1	forward	GCTTCCGGCTAAGAACGGT
	reverse	TTGCGGACATAGTTGAGGAGT
GAD2	forward	ATTGGGAATTGGCAGACCAAC
	reverse	TTGAAGTATCTAGGATGCCCTGT
GABBR1	forward	CTGTGCCCGTCAAAAACCTG
	reverse	TTCTTCCCAAAGAGACGCTCC

PVALB	forward	AAGAGTGCGGATGATGTGAAG
	reverse	GCCTTTTAGGATGAATCCCAGC
SST	forward	ACCCAACCAGACGGAGAATGA
	reverse	GCCGGGTTTGAGTTAGCAGA
CALB2	forward	AGCGCCGAGTTTATGGAGG
	reverse	TGGTTTGGGTGTATTCCTGGA
ChAT	forward	CAGCCCTGCCGTGATCTTT
	reverse	TGTAGCTGAGTACACCAGAGATG
SLC18A3	forward	TTCGCCTCTACAGTCCTGTTC
	reverse	GCTCCTCCGGGTACTTATCG
ACHE	forward	GGGTGGTAGACGCTACAACC
	reverse	GTGCCCTCAAAACCTGGGTAT
NGFR	forward	CCTACGGCTACTACCAGGATG
	reverse	CACACGGTGTTCTGCTTGT
SLC5A7	forward	AAACCTATGCGTTCAAAGGGG
	reverse	GCAGGAATAAACAGGAGTCCG
ISL1	forward	GCGGAGTGTAATCAGTATTTGGA
	reverse	GCATTTGATCCCGTACAACCT
LHX3	forward	CAGTATTTCCGCAACATGAAGC
	reverse	GCTCCCGTAGAGGCCATTG
LHX4	forward	CACTGCTTTGCTTGCATCATC
	reverse	GGCTGTCTCGTAGTCTTCCTT
Exogenous *FOXA2* gene in FOXA2-iOE cells	forward	CACACCACAGAAGTAAGGTTCC
	reverse	TCCCGGCCCATTATGAACTC

Table S5. List of regents and resources used in this study.

REAGENT or RESOURCE	SOURCE	IDENTIFIER
**Antibodies**		
Goat polyclonal anti-SOX1	R&D system	Cat#AF3369;<br>RRID: AB_2239879
Mouse monoclonal anti-NKX2.2	DSHB	Cat#74.5A5;<br>RRID: AB_531794
Goat polyclonal anti-FOXA2	R&D system	Cat#AF2400;<br>RRID: AB_2294104
Mouse monoclonal anti-FOXA2	Santa Cruz Biotechnology	Cat#sc-101060;<br>RRID: AB_1124660
Mouse monoclonal anti-β-Tubulin III	Sigma-Aldrich	Cat# T8660;<br>RRID: AB_477590
Mouse monoclonal anti-β-Tubulin	Yeasen Biotech	Cat# 30301ES40;<br>RRID: N/A
Mouse monoclonal anti-Netrin1	Abcam	Cat# ab126729;<br>RRID: AB_11131145
Rabbit polyclonal anti-NKX6-1	Sigma-Aldrich	Cat# HPA036774;<br>RRID: AB_10673664
Goat polyclonal anti-PHOX2B	R&D Systems	Cat# AF4940;<br>RRID: AB_10889846
Rabbit polyclonal anti-OLIG2	Millipore	Cat# AB9610; RRID: AB_570666
Rabbit polyclonal anti-Substance P	ImmunoStar	Cat# 20064; RRID: AB_572266
Rat monoclonal anti-CORIN	R&D Systems	Cat# MAB2209;<br>RRID: AB_2082224
Rat monoclonal anti-HOXB4	DSHB	Cat# I12 anti-Hoxb4;<br>RRID: AB_2119288
Rabbit polyclonal anti-5-HT	lmmunoStar	Cat#20080; RRID: AB_572263
Goat polyclonal anti-5-HT	Abcam	Cat# ab66047; RRID: AB_1142794
Rabbit polyclonal anti-TPH2	Thermofisher	Cat#PA1-778;<br>RRID: AB_2207687
Mouse monoclonal anti-Gata3	R&D systems	Cat#MAB6330;<br>RRID: AB_10640512
Mouse monoclonal anti-GFP	Abcam	Cat#AB1218;<br>RRID: AB_298911

Rabbit polyclonal anti-GFP	Beyotime	Cat#AG279;<br>RRID: AB_2893351
Mouse monoclonal anti-ASCL1	BD Biosciences	Cat#556604;<br>RRID: AB_396479
Rabbit polyclonal anti-TH	chemicon	Cat#AB152;<br>RRID: AB_390204
Cy™3 AffiniPure Donkey Anti-Goat IgG (H+L)	Jackson ImmunoResearch	Cat#705-165-003;<br>RRID:AB_2340411
Fluorescein (FITC) AffiniPure Donkey Anti-Goat IgG (H+L)	Jackson ImmunoResearch	Cat#705-095-003;<br>RRID:AB_2340400
Fluorescein (FITC)-conjugated AffiniPure Donkey Anti-Mouse IgG (H+L)	Jackson ImmunoResearch	Cat#715-095-150;<br>RRID:AB_2340792
Cy™3-conjugated AffiniPure Donkey Anti-Mouse IgG (H+L)	Jackson ImmunoResearch	Cat#715-165-15;<br>RRID:AB_2340813
Fluorescein (FITC)-conjugated AffiniPure Donkey Anti-Rabbit IgG (H+L)	Jackson ImmunoResearch	Cat#711-095-152;<br>RRID:AB_2315776
Cy™3-conjugated AffiniPure Donkey Anti-Rabbit IgG (H+L)	Jackson ImmunoResearch	Cat#711-165-152;<br>RRID:AB_2307443
Cy™3 AffiniPure Donkey Anti-Rat IgG (H+L)	Jackson ImmunoResearch	Cat#712-165-150;<br>RRID: AB_2340666
Peroxidase-Conjugated Goat Anti-Rabbit IgG (H+L)	Yeasen Biotech	Cat# 33101ES60;<br>RRID:AB_2922405
Peroxidase-Conjugated Goat Anti-Mouse IgG (H+L)	Yeasen Biotech	Cat# 33201ES60;<br>RRID:AB_10015289
**Chemicals, peptides, and recombinant proteins**		
SB 431542	TargetMol	Cat#T1726;<br>CAS 301836-41-9
DMH1	TargetMol	Cat#T1942;<br>CAS 1206711-16-1
CHIR 99021	TargetMol	Cat#T2310;<br>CAS 252917-06-9
Purmorphamine	TargetMol	Cat#T1810;<br>CAS 483367-10-8
Y-27632 dihydrochloride	TargetMol	Cat#T1725;<br>CAS 129830-38-2
DAPT	TargetMol	Cat#T6202;<br>CAS 208255-80-5

Retinoic acid	Sigma-Ardrich	Cat#R2625; CAS 302-79-4
Ro 41-5253	Sigma-Ardrich	Cat#SML0573; CAS 144092-31-9
LE135	Sigma-Ardrich	Cat#SML0809; CAS 155877-83-1
LY 2955303	Med Chem Express	Cat#HY-107765; CAS 1433497-19-8
AM580	TargetMol	Cat#T5854; CAS 102121-60-8
Doxycycline	TargetMol	Cat#T1140; CAS 10592-13-9
Cyclopamine	TargetMol	Cat#T2825; CAS 4449-51-8
Escitalopram Oxalate	TargetMol	Cat#T6493; CAS 219861-08-2
FGF4	Novoprotein	Cat#CR08
GDNF	PeproTech	Cat#450-10
BDNF	PeproTech	Cat#450-02
TGFβ3	Novoprotein	Cat#CJ44
IGF1	PeproTech	Cat#100-11
bFGF	PeproTech	Cat#100-18B
2-Mercaptoethanol	Sigma-Ardrich	Cat#M3148
poly-l-ornithine	Sigma-Ardrich	Cat#P3655
Vitamin C	Sigma-Ardrich	Cat#A4403
Matrigel	Corning	Cat#354277
mTeSR™1	Stemcell Technologies	Cat#85850
TrypLE	Gibco	Cat#12604021
DMEM/F12	Gibco	Cat#11330-032
Neurobasal	Gibco	Cat#21103049
N2	Gibco	Cat#17502048
B27	Gibco	Cat#12587010
Non-Essential amino acids (NEAA)	Gibco	Cat#11140050
GlutaMAX	Gibco	Cat#35050061
Knockout™ SR	Gibco	Cat#10828028
Laminin	Gibco	Cat#23017015
**Critical commercial assays**		
Serotonin high sensitive ELISA kit	IBL International GmbH	Cat#RE59141
Kapa genomic DNA extraction kit	Kapabiosystems	Cat#KK7102

**Deposited data**		
Raw and analyzed data	This study	GEO:GSE232830
**Experimental models: Cell lines**		
Human: H9 embryonic stem cells	WiCell Research Institute	WA01; RRID: CVCL_9771
Human: H1 embryonic stem cells	WiCell Research Institute	WA01; RRID: CVCL_9771
Human: induced pluripotent stem cells (BJTTHi001-A/ZSSY 001)	Nuwacell	RC01001-A; RRID: CVCL_A8HA
Human: FKO1 stem cell line	This study	N/A
Human: FKO2 stem cell line	This study	N/A
Human: FOXA2-iOE stem cell line	This study	N/A
Human: FOXA2-tracing stem cell line[10]		N/A
Human: FKO TPH2^{EGFP} stem cell line	This study	N/A
Human: H9 TPH2^{EGFP} stem cell line[1]		N/A
**Recombinant DNA**		
pENTER-FOXA2	Vigene Biosciences	plasmid #CH893992; RRID: N/A
pX330-U6-Chimeric_BB-CBh-hSpCas9	Addgene	Addgene plasmid #42230; RRID: Addgene_42230
pSpCas9(BB)-2A-Puro (PX459)	Addgene	Addgene plasmid #48139; RRID:Addgene_48139
AAVS1-TRE3G-EGFP	Addgene	Addgene plasmid #52343; RRID: Addgene_52343
pUC19	Addgene	Addgene plasmid #50005; RRID: Addgene_50005
PX330-sgRNA	N/A	N/A
PX459-sgRNA	N/A	N/A
TPH2-T2A-EGFP	N/A	N/A
sgT2-Cas9[1]		N/A
pUC19-Stop-SV40-Neo	N/A	N/A
**Software and algorithms**		

GraphPad PRISM 6	GraphPad	http://www.graphpad.com; RRID:SCR_002798
ImageJ	NIH	https://imagej.nih.gov/ij/; RRID:SCR_003070
pClamp10.2	Molecular Devices	http://www.moleculardevices.com RRID:SCR_011323
Origin	OriginLab	http://www.originlab.com RRID:SCR_014212

References:

[1] T. Xu, J. Duan, Y. Li, G. Wang, S. Li, Y. Li, W. Lu, X. Yan, Y. Ren, F. Guo, L. Cao, J. Lu, *Stem Cell Rep*. **2022**, *17*, 2365.

[2] B. W. Okaty, N. Sturrock, L. Y. Escobedo, Y. Chang, R. A. Senft, K. A. Lyon, O. V. Alekseyenko, S. M. Dymecki, *Elife*. **2020**, *9*.

[3] J. D. Welch, V. Kozareva, A. Ferreira, C. Vanderburg, C. Martin, E. Z. Macosko, *Cell*. **2019**, *177*, 1873.

[4] C. A. Fasano, S. M. Chambers, G. Lee, M. J. Tomishima, L. Studer, *Cell Stem Cell*. **2010**, *6*, 336.

[5] J. Lu, X. Zhong, H. Liu, L. Hao, C. T. Huang, M. A. Sherafat, J. Jones, M. Ayala, L. Li, S. C. Zhang, *Nat. Biotechnol.* **2016**, *34*, 89.

[6] B. W. Okaty, M. E. Freret, B. D. Rood, R. D. Brust, M. L. Hennessy, D. DeBairos, J. C. Kim, M. N. Cook, S. M. Dymecki, *Neuron*. **2015**, *88*, 774.

[7] B. W. Okaty, K. G. Commons, S. M. Dymecki, *Nat. Rev. Neurosci.* **2019**, *20*, 397.

[8] J. Ren, A. Isakova, D. Friedmann, J. Zeng, S. M. Grutzner, A. Pun, G. Q. Zhao, S. S. Kolluru, R. Wang, R. Lin, P. Li, A. Li, J. L. Raymond, Q. Luo, M. Luo, S. R. Quake, L. Luo, *Elife*. **2019**, *8*.

[9] K. W. Huang, N. E. Ochandarena, A. C. Philson, M. Hyun, J. E. Birnbaum, M. Cicconet, B. L. Sabatini, *Elife*. **2019**, *8*.

[10] Z. Chen, X. Ren, X. Xu, X. Zhang, Y. Hui, Z. Liu, L. Shi, Y. Fang, L. Ma, Y. Liu, D. Terheyden-Keighley, L. Liu, X. Zhang, *Stem Cell Rep*. **2018**, *11*, 1257.
